# Paper-Based In-Situ Gold Nanoparticle Synthesis for Colorimetric, Non-Enzymatic Glucose Level Determination

**DOI:** 10.3390/nano10102027

**Published:** 2020-10-14

**Authors:** Tomás Pinheiro, João Ferrão, Ana C. Marques, Maria J. Oliveira, Nitin M. Batra, Pedro M. F. J. Costa, M. Paula Macedo, Hugo Águas, Rodrigo Martins, Elvira Fortunato

**Affiliations:** 1 CENIMAT/i3N, Materials Science Department, Faculdade de Ciência e Tecnologia–Universidade Nova de Lisboa, 2829-516 Lisbon, Portugal; tp.pinheiro@campus.fct.unl.pt (T.P.); j.ferrao@campus.fct.unl.pt (J.F.); accm@campus.fct.unl.pt (A.C.M.); mj.oliveira@campus.fct.unl.pt (M.J.O.); hma@fct.unl.pt (H.Á.); rfpm@fct.unl.pt (R.M.); 2 Physical Science and Engineering Division, King Abdullah University of Science and Technology (KAUST), Thuwal 23955-6900, Saudi Arabia; nitinkumar.batra@kaust.edu.sa (N.M.B.); pedro.dacosta@kaust.edu.sa (P.M.F.J.C.); 3 CEDOC, Chronic Disease Research Centre, NOVA Medical School, Faculdade de Ciências Médicas, Universidade NOVA de Lisboa, Campo Mártires da Pátria, 1150-190 Lisbon, Portugal; paula.macedo@nms.unl.pt; 4 Education and Research Centre, APDP-Diabetes Portugal (APDP-ERC), 1250-203 Lisbon, Portugal

**Keywords:** diabetes, glucose, in-situ synthesis, gold nanoparticles, enzyme-free, colorimetric, paper-based platform

## Abstract

Due to its properties, paper represents an alternative to perform point-of-care tests for colorimetric determination of glucose levels, providing simple, rapid, and inexpensive means of diagnosis. In this work, we report the development of a novel, rapid, disposable, inexpensive, enzyme-free, and colorimetric paper-based assay for glucose level determination. This sensing strategy is based on the synthesis of gold nanoparticles (AuNPs) by reduction of a gold salt precursor, in which glucose acts simultaneously as reducing and capping agent. This leads to a direct measurement of glucose without any enzymes or depending on the detection of intermediate products as in conventional enzymatic colorimetric methods. Firstly, we modelled the synthesis reaction of AuNPs to determine the optical, morphological, and kinetic properties and their manipulation for glucose sensing, by determining the influence of each of the reaction precursors towards the produced AuNPs, providing a guide for the manipulation of nucleation and growth. The adaptation of this synthesis into the developed paper platform was tested and calibrated using different standard solutions with physiological concentrations of glucose. The response of the colorimetric signals obtained with this paper-based platform showed a linear behavior until 20 mM, required for glycemic control in diabetes, using the Red × Value/Grey feature combination as a calibration metric, to describe the variations in color intensity and hue in the spot test zone. The colorimetric sensor revealed a detection limit of 0.65 mM, depending on calibration metric and sensitivity of 0.013 AU/mM for a linear sensitivity range from 1.25 to 20 mM, with high specificity for the determination of glucose in complex standards with other common reducing interferents and human serum.

## 1. Introduction

Glucose is the primary energy source of cells and acts as a metabolic intermediate, being transported through the bloodstream of organisms [1,2]. The normal levels of glucose in human blood range from 3.8 to 6.9 mM (68–124 mg/dL), approximately, although these levels may change during the day, being lower upon fasting and reaching a peak in postprandial states [3]. Both high and low glycemic levels are detrimental for health status, for example, a level below 2.8 mM (50 mg/dL) after fasting or following exercise is considered an hypoglycemic event [4]. The incidence of glucose levels outside the normal range may indicate the presence of health conditions, such as diabetes mellitus [1]. Diabetes mellitus is a chronic disease characterized by persistently high glucose levels and caused by the incapacity of the pancreas to produce sufficient insulin (type I diabetes) or by its inability to promote glucose uptake (type II diabetes) [5]. For diabetes management, blood glucose concentration should be strictly controlled and below 10 mM (180 mg/dL) according to the American Diabetes Association [6], otherwise it can lead to serious complications including diabetic retinopathy, kidney failure, strokes, heart attacks, fatty liver disease, high blood pressure, blindness, and coma [7]. 

Although there is no cure for diabetes, blood glucose monitoring combined with appropriate medication can enhance treatment efficacy, alleviate symptoms, and diminish the complications of the condition [8,9]. Nowadays, the main methods for glucose sensing and monitoring are invasive and based mostly on electrochemical detection of either hydrogen peroxide as a by-products or electrons released upon oxidation of glucose via the glucose oxidase enzyme. This is the benchmark in glucose detection in the point-of-care, with test strips that include sample processing and metabolite determination in easy-to use, small, and fast fashion [10]. More recently, CGM (Continuous Glucose Monitoring) devices have been employed for glucose detection, providing a continuous and real-time monitoring of glucose and alerting patients in case of sudden variation or approach to undesirable glucose levels [5,11]. Nonetheless, the World Health Organization established guidelines for the development of diagnostic point-of-care tests, which are summarized under the acronym ASSURED (Affordable, Sensitive, Specific, User-friendly, Rapid and robust, Equipment-free, and Delivered to those in need) [12]. Paper is considered a viable alternative to serve as a substrate for disposable diagnostic devices, not only due to its biodegradability and biocompatibility, but also because of its availability and cheap manufacturing costs, in every part of the world [12,13]. Moreover, its color, usually white, serves as a good background in platforms for colorimetric detection, with the advantage that color changes can be detected by visual inspection [14]. Current methods for detection and quantification of glucose using paper-based devices are based on decomposition of glucose by the action of glucose oxidase (GOx) or enzyme mimicking materials, producing hydrogen peroxide, followed by a catalytic reaction with H_2_O_2_ and the use of peroxidase to oxidize colorimetric indicators or produce detection potentials, resulting in signals that are proportional to glucose concentration in the sample [1,2,3]. However, all the above glucose detection methods are indirect or require enzymatic reactions. In this setting, enzymes present several shortcomings, mainly related to purification and subsequent applications and immobilization in sensing platforms, associated with inherent stability issues related to the chemical environment surrounding the electrocatalytic reactions, mainly temperature, humidity, and pH, that jeopardize their high selectivity. Furthermore, the necessary use of bienzymatic systems lead to stringent environmental conditions for optimal performance, influence shelf life, and call for rigid storage requirements of platforms [15,16]. Thus, non-enzymatic sensing approaches have been proposed as a beneficial alternative to replace enzymes and the necessary enzymatic bioactivity by more reliable, reproducible, and simple materials, showing great promise for high performance sensor development [16,17,18]. Various approaches have been presented when developing non-enzymatic sensing mechanisms, with the use of nanostructured materials standing out due to their disruptive electrical, chemical, optical, and catalytic properties, that are very advantageous for application in glucose sensing [19,20]. The use of in-situ synthesis of nanomaterials for colorimetric sensing has gained much attention due to its simplicity, ease of operation, and good sensitivity [21]. Some optical, colorimetric glucose sensing mechanisms based on noble metal nanoparticles have been presented in the literature, but most rely on pre-functionalization of particles or complementary use of enzymes or other catalytic reactions for the production of color signals, instead of using direct reactions between glucose and the precursor reagents, and there is yet to be such approach translated into a paper-based platform [22,23,24,25,26,27]. Nanostructured gold has also been applied in electrochemical sensing as a means of improving sensors catalytic capabilities, both in conjugation with GOx or for the development of non-enzymatic sensors [28,29,30,31,32]. However, these require more complex setups, both in terms of manufacture and use, hindering their portability and sustainability, when compared to a paper-based, colorimetric approach.

Herein, we report, for the first time, the development of a novel, rapid, disposable, inexpensive, enzyme-free, and colorimetric paper-based platform for glucose sensing in the physiologically relevant range (1.25–20 mM). The platform construction was performed using Lab-on-Paper technology, with definition of hydrophilic test zones on paper, delimited by hydrophobic barriers, formed by printing wax patterns on the paper surface followed by diffusion of wax layers throughout the paper’s thickness [12,13,33,34,35]. Regarding the glucose detection method, it is based on the synthesis of gold nanoparticles (AuNPs) by reduction of a gold salt in the presence of NaOH, required for hydrolysis of gold complex ions, in which glucose is the reducing agent [36,37,38]. When the concentration of glucose is altered, differently sized AuNPs are produced: Lower glucose concentrations correspond to larger nanoparticles, whereas higher glucose concentrations are associated to smaller nanoparticles. On the paper platform, it results on the display of a red color, that is more intense and varies its hue, when glucose concentration increases. These color changes were analyzed by visual inspection and color space analysis of Red, Green, Blue (RGB), Hue, Saturation, and Value (HSV), and three-dimensional cartesian coordinate system (XYZ) of images obtained with a commercial scanner, to obtain a semi-quantitative, simple and efficient approach for colorimetric, glucose sensing by the reducing and capping effect of this metabolite towards the gold salt. With this approach, there is no dependence of any reaction products used as mediators in conventional enzymatic sensing approaches, resulting in a direct glucose dependent color signal obtained without the use of any enzymes, showing the potential for enzyme-free, colorimetric glucose level determination in the point-of-care.

## 2. Materials and Methods

### 2.1. Synthesis of AuNPs in Solution

Firstly, solutions of each precursor reagent were prepared using a Milli-Q water filtration system 18.2 Ω (Millipore Corporation, Billerica, MA, USA). D-Glucose (Sigma-Aldrich, St. Louis, MO, USA) solutions were prepared to each respective concentration (1.25–50 mM) using deionized water. Moreover, solutions of sodium hydroxide (NaOH, Fisher Scientific, Loughborough, UK) and tetrachloroauric acid trihydrate (HAuCl_4_·3H_2_O, Acros Organics, NJ, USA) were also prepared. HAuCl_4_·3H_2_O solutions of each concentration were added in 500 μL aliquots to eppendorf tubes (or microcentrifuge tubes), subsequently placed on a vortex mixer (VELP Scientifica, Usmate Velate, Italy). While vortexing, 10 μL of NaOH solution with desired concentration were added to promote gold complex ion hydrolysis. After 1 min, 500 μL of D-glucose solution were added to the vortexing solution, inducing the reduction of gold salt complex ions and the formation of AuNPs. For the study of reaction kinetics by in-situ synthesis of AuNPs at the UV-Vis spectrophotometer, this procedure was carried in quartz cuvettes placed inside the device holder. HAuCl_4_ and NaOH were mixed in a cuvette and hydrolysis took place for 1 min. After 1 min, measures were started upon the introduction of reductant and start of AuNPs’ formation. All experiments were performed at room temperature (25 °C), to reduce the influence of this variable on the synthesis of AuNPs.

### 2.2. AuNPs Characterization

The colorimetric changes resulting from the synthesis of AuNPs in solution were analyzed by UV-Vis spectrophotometry. LSPR measurements were taken using a multifunctional microplate reader (Tecan Spark 10M, Tecan, Männedorf, Switzerland) equipped with a Xenon-flash lamp and a high-speed monochromator. Two hundred microliters of each AuNPs’ colloidal suspension was deposited in the wells of a 96-well, transparent, flat-bottomed, polystyrene microplate and the measurements were performed in the wavelength range between 400 and 800 nm.

In-situ UV-Vis measures, for the study of reaction kinetics, were performed using a Thermo Scientific Evolution 300 UV–Vis spectrophotometer (Thermo Fisher Scientific, Waltham MA, USA), to allow for time-resolved measures (Abs_t_). After the precursor reagents are mixed in quartz cuvettes placed in the spectrophotometer holder, measures are started, to follow the evolution of peak absorbance wavelength, corresponding to the plasmon resonance wavelength, over time.

Transmission electron microcopy (TEM) studies were carried out using a transmission electron microscope (ThermoFisher Scientific Inc, TITAN C-Twin, operated at 300 kV), and samples were prepared using Cu grids covered with a thin-carbon film, which is characterized by its transparency and resistance to the electron beam. The thin-carbon TEM grids were drop-casted with the solutions containing the AuNPs and then allowed to dry naturally, at room temperature. The carbon films were immersed in the solutions containing the AuNPs, allowed to dry at room temperature for a few minutes, and then placed on the Cu grids for further TEM analysis.

### 2.3. Paper-Based Platform Fabrication

The first stage for the paper substrates preparation was the creation of test zones by patterning of hydrophobic barriers. Whatman no. 1 chromatography paper substrate sheets (570 × 460 mm, Whatman International Ltd., Floram Park, NJ, USA) were cut into A5 standard format (210 × 148 mm) and fed to the manual feed tray of a commercial solid ink printer (Xerox ColorQube 8570, Xerox Corporation, Norwalk, CT, USA) designed to print a wax-based ink. All the prototypes used in this work were designed using Adobe Illustrator software (Adobe Systems Software, Ireland). The papers with printed patterns were then placed on a hot plate (Heidolph MR HeiTec, Schwabach, Germany) at 120 °C for about 2 min, allowing the wax to melt and spread vertically through the whole thickness of the paper, creating the desired hydrophobic barriers (Scheme S1).

### 2.4. Synthesis of AuNPs at Paper Substrate

The synthesis of AuNPs on paper substrate was performed via in-situ gold salt reduction by glucose, based on the previously described synthesis of AuNPs in aqueous medium. The same reagents were used as in the synthesis in solution, although the respective volumes, concentrations, and order of reagent introduction was altered to achieve a lab-on-paper approach. Each reaction zone (Ø = 7 mm) was impregnated with 2.5 μL of HAuCl_4_ solution and the paper platforms were dried at room temperature. Then, 2.5 μL of NaOH solution were added to each well and allowed to dry at room temperature for 10 min, promoting hydrolysis of gold(III) chloride complex ions in the paper substrate. Finally, 2.5 μL of D-Glucose solution with desired concentration were added to each well, leading to the formation of AuNPs on paper. The drying process was continued for a few more minutes to ensure reaction completion. All experiments were performed at room temperature (25 °C), to reduce the influence of this variable on the synthesis of AuNPs.

### 2.5. Data Acquisition and Colorimetric Analysis

The colorimetric paper-based results were recorded with a digital scanner (All-in-One Printer 1050A HP, Hewlett-Packard Development Company, L.P., Palo Alto, CA, USA) with 600 dpi resolution and the digitalized data were then analyzed without further manipulation, using a custom image processing software, for analysis of various color space features, more specifically, RGB (Red, Green, Blue), HSV (Hue, Saturation, and Value), and XYZ. Crops of reaction zones (50 × 50 pixels) are automatically drawn out and features extracted from pixels, followed by the computation of the mean values of each feature in the crop. Mean feature values were correlated to glucose concentrations, with outlier detection criteria of values above 3σ.

### 2.6. Testing Against Interfering Agents

Testing against common reducing agents in biological solutions (fructose, galactose, ascorbic acid, glutathione, and lipoic acid) [39] was carried in the paper substrate, to assess if they influence the direct reduction of gold salt by glucose. To do so, solutions with twice the desired concentrations of interfering agents were prepared in MilliQ water and mixed with glucose solutions. Posteriorly, the synthesis was carried as previously presented. Additionally, solutions containing only the interfering agents, without glucose, were used to carry the synthesis, to show if the interfering agents alone could produce gold nanoparticles.

### 2.7. AuNPs Characterization at Paper Substrate

Spectral profiles of AuNPs synthesized in-situ at the paper substrate were collected by cutting paper-disks with AuNPs, that were placed at the wells of a 96-well, transparent, flat-bottomed, polystyrene microplate, with measurements performed using the same methodology as for colloidal suspensions, with a wavelength range between 400 and 800 nm. Nanoparticles’ morphology was characterized by SEM-EDS (Carl Zeiss AURIGA Crossbeam SEM-FIB, Oberkochen, Germany) using an accelerating voltage of 2 keV with an aperture size of 30 μm and a working distance between the sample and the SEM column of 5.8 mm. The crystallographic structure of the nanoparticles was determined by XRD (X’Pert Pro MPD, PANalytical, Almelo, The Netherlands) with a CuKα target and a wavelength of 1.5406 Å. The diffraction pattern was acquired at angles (2θ) between 10° and 90° with a step of 0.03°, in the continuous mode and operating at 45 kV and 40 mA. The presence of AuNPs on the paper was proved by SERS (Surface-Enhanced Raman Spectroscopy). Tetraethylrhodamine hydrochloride (Rhodamine 6G-R6G) was selected as a SERS probe because of its very intense and distinct Raman signals and used without further purification. According to a previous work [40], SERS samples were prepared by dropping 2 μL of a 1 × 10^−6^ M R6G (Sigma-Aldrich, St. Louis, MO, USA) solution on the paper substrate, while a 1 × 10^−3^ M R6G solution was used in glass as the reference control. Raman measurements were performed in a LabRam 300 Horiba Jobin Yvon spectrometer (Horiba, Kyoto, Japan) equipped with an air-cooled CCD detector and a HeNe laser operating at 1750 μW of 632.81 nm laser excitation. Spectral analysis was performed with the curve-fitting program Peakfit v4.12 (Seasolve Software Inc., San Jose, CA, USA). After linear baseline subtraction, Lorentzian decomposition of spectra was performed to identify peak parameters (height, area, center, and width) associated with selected Raman lines and then the average SERS enhancement factors (EF) were calculated.

### 2.8. Colorimetric Sensing in Complex Samples

A validation of the presented method was carried using complex solutions, more specifically, human serum, obtained from a healthy donor. Considering the matrix effect of blood samples and its derivatives, a protein precipitation protocol was used for extraction of proteins, as they influence the formation of AuNPs. Spiked serum samples with desired concentrations of glucose were mixed with different volumes of ethanol (1:1, 1:2 and 1:3 ratios of serum:ethanol, resulting in 50%, 33%, and 25% diluted serum samples). Ethanol leads to the precipitation of proteins in samples, that were then centrifuged at 6000 rpm for 10 min, with the resulting supernatant (without ethanol extraction) being used to carry the synthesis. Protein precipitation and removal efficiency was tested using the colorimetric Biuret reagent. The production of the Biuret reagent was the following: 0.4 g of NaOH was dissolved in 40 mL deionized water, followed by dissolving 0.45 g of sodium potassium tartrate. Posteriorly, 0.15 g of copper sulfate was dissolved in this mixture, followed by dissolution of 0.45 g of potassium iodide. Finally, the mixture volume was made to 50 mL by addition of deionized water.

## 3. Results

### 3.1. AuNPs Synthesis Mechanisms through Reduction and Capping by Glucose

In the green synthesis of AuNPs promoted by glucose, the aldehyde functional group is oxidized, being responsible for electron donation, reducing Au(III) ion from HAuCl_4_ to Au(0) oxidation state. This leads to formation of Au seeds in solution, whose size corresponds to a color shift depending on glucose concentration in the system, as previously reported [36,41,42].

The first step in the production of AuNPs by glucose reduction of the gold salt precursor is the mixing of an aqueous solution containing AuCl_4_^−^ ions with sodium hydroxide (NaOH), in order to adjust pH and cause the hydrolysis of Au(III) chloride complex ions, which is governed by the following chemical equation:

(1) $${{[\mathrm{AuCl}}_{4}]}^{-}{\;+\;\mathrm{nOH}}^{-}\overset{\;{\;k}_{h}\;}{\to }{\;[\mathrm{AuCl}}_{4-n}{\left(\mathrm{OH}\right)}_{n}{]}^{-\;}{+\;\mathrm{nCl}}^{-}$$

where n is the number of Cl^−^ ligands exchanged by [OH]^−^ ions (n = 1, 2, 3, 4). Previous studies presented in the literature have shown that gold species distribution is dependent on pH, with a higher rate of exchange of Cl^−^ by OH^−^ occurring for higher pH values as well as initial concentration of gold and chloride, as variations in ionic strength occur [43]. However, at room temperature, the main species of gold complex ions formed is AuCl_3_(H_2_O), which is quickly dissociated to [AuCl_3_(OH)]^−^ [44].

Upon introduction of D-glucose aqueous solution into the hydrolyzed precursor solution, the reduction process starts, with the base facilitating the opening of the sugar ring by abstraction of the α-proton in the oxygen atom, leading to oxidation of glucose to gluconic acid [38]. After a certain amount of time, colloidal solutions show strong coloration ranging from red to purple/blue, depending on the reaction parameters. This is caused by the reduction of Au(III) ions by glucose, a process governed by the following chemical equation:

(2) $${\;[\mathrm{AuCl}}_{3}{\left(\mathrm{OH})\right]}^{-}{\;+\;C}_{6}H_{12}O_{6}\overset{\;}{\to }{\;\mathrm{Au}}_{\mathrm{colloid}}\;+\;\mathrm{products}$$

Although Au(III) ions are the most common complexes formed after hydrolysis, it has been shown in literature that Au(I) complexes are present as intermediates between the hydrolysis and the formation of gold nanoclusters, meaning the nucleation and growth steps are subsequent to transition from Au(III) to Au(I) oxidation states, and subsequent formation of gold seeds in the solid state [45]. Thus, by application of the Finke–Watzky theory for nucleation and growth [46] towards this AuNPs production method, the synthesis of solid gold seeds and their growth is governed by the following equations:

(3) $${\mathrm{Au}(I)\;+\;C}_{6}H_{12}O_{6}\;\overset{\;{\;k}_{1}\;}{\to }\;\mathrm{Au}\;+\;\mathrm{products}$$

(4) $${\mathrm{Au}(I)\;+\;C}_{6}H_{12}O_{6}\;+\;\mathrm{Au}\;\overset{\;{\;k}_{2}\;}{\to }\;2\mathrm{Au}\;+\;\mathrm{products}$$

Equation (3) corresponds to slow continuous nucleation of gold atoms reduced by glucose, which in turn leads to fast autocatalytic growth, as Au atoms in the solid state further promote the reduction by serving as nucleation sites, represented by Equation (4). From these chemical equations, it is possible to derive the kinetic formulas describing these mechanisms, which are used to fit and study the experimental kinetic data [45,46,47].

(5) $$C_{\mathrm{Au}\;}{=C}_{0,\mathrm{Au}\left(I\right)}\left(1\;-\;\frac{{k_{1}+k_{2}C}_{0,\mathrm{Au}\left(I\right)}}{{k_{2}C}_{0,\mathrm{Au}\left(I\right)}+{k_{1}e}^{\left({k_{1}+k_{2}C}_{0,\mathrm{Au}\left(I\right)}\right)t}}\right)$$

Equation (5) is used to derive Equation (6), showing the relationship between UV-Vis spectrophotometry absorbance experimental measures and concentration, as χ = *C*_Au_/*C*_0,Au(I)_ = Abs_t_/Abs_max_:

(6) $${\mathrm{Abs}}_{t}{\;=\;\mathrm{Abs}}_{\max}\left(1\;-\;\frac{k_{1}\;+{k_{2}}^{\prime }}{k_{1}e^{\left(k_{1}{+k}_{2}\right)t}\;+{k_{2}}^{\prime }}\right)$$

These equations are used to fit the sigmoidal shape exhibited by experimental kinetic curves and to determine the correspondent rate constants and how they are influenced by reaction parameters.

#### 3.1.1. Effect of Reaction Parameters towards Optical and Kinetic Properties

Considering the previously presented model for studying the kinetics of AuNPs formation through gold(III) complex ions reduction by glucose, the effects of each reaction parameter were examined. The following reaction parameters were studied: (i) Gold salt precursor concentration; (ii) reaction system volume; (iii) NaOH concentration/pH; and (iv) glucose concentration. Other relevant variables, such has system temperature, also influence optical and kinetic properties of the synthesis [45], but were not studied in this work, as a uniform temperature control method during synthesis and kinetic measurements was not achieved.

The influence of initial gold salt concentrations was studied and results are presented in Figure S1A,B. Different initial concentrations of precursor were applied to the synthesis (0.2–1 mM), maintaining the concentration of reductant and NaOH for all repetitions (10 mM and 0.5 M, respectively). As noted in Figure S1A, the monitoring of absorbance at LSPR wavelength (λ_max_) shows that the increase of gold salt initial concentration leads to a variation in the size range of particles. We found that λ_max_ and width of the optical profile decrease when more gold salt complex ions are introduced, meaning the size and polydispersity of particles are smaller. This parameter can then be used to reach a λ_max_ variation range that allows for a colorimetric response to samples containing glucose in the physiological range. Time-resolved variation of absorbance at λ_max_ showed that kinetic curves present a sigmoidal shape for each of the tested conditions. The Abs_t_/Abs_max_ normalized curves, presented in Figure S1B, have three characteristic stages related to the synthesis dynamics of these colloidal systems: (i) Induction stage, (ii) fast growth stage characterized by the steep increase in absorbance, and (iii) saturation stage, where there is the completion of the reaction and precursors have been consumed. Time features, obtained by fitting the experimental curves with Equation (6), change with reaction conditions (corresponding rate constants are presented in Table S1). As it can be seen, the general time frame of AuNP formation tends to increase, as the steep increase in absorbance at λ_max_ happens at subsequently longer periods after introduction of the reducing agent. For the lowest initial HAuCl_4_ concentration, the start of steep increase in absorbance happens around 50 s, with a growth rate constant of k_2_ = 0.38, while for the highest HAuCl_4_ concentration, this time delay increases to close to double, with k_2_ = 0.19. This means that the length for incubation, where reduction of Au(III) complex ions occurs and nuclei are formed, increases. Consequently, there seems to be a critical concentration of gold atoms or seeds that need to be formed in the colloidal system, before the autocatalytic growth starts, which is correlated to the increasing amount of initial gold salt concentration.

Regarding reaction system volume, different initial gold salt precursor solution volumes (250–1250 µL) were applied, maintaining the precursor concentration (0.2 mM), as well as the concentration and volume of reductant and NaOH for precursor hydrolysis (500 µL, 50 mM and 10 µL, 0.5 M, respectively). Results for the optical features of the obtained AuNPs are presented in Figure S1C. By increasing the initial volume of gold salt precursor, there is a rise in the absorbance of the colloidal suspensions, without a significant variation of LSPR wavelength (λ_max_ = 545 ± 2 nm), apparently due to the formation of higher amounts of particles, as more gold salt precursor is available to be reduced. Regarding the kinetic curves, presented in Figure S1D, the sigmoidal shape of the kinetic curve is maintained, with the increase in system volume leading to a general increase in the time for growth to begin, resulting in a controllable time delay for the start of AuNPs’ formation, paired with a decrease of growth rate of AuNPs (given by the derivative of the kinetic curves in Figure S2). Regarding the kinetic rate constants, both the nucleation (k_1_) and growth (k_2_) rate constants shift to lower values, as can be seen in Table S2.

The effect of initial NaOH concentration and thus, the reaction pH, upon introduction of D-glucose, was assessed, with different concentrations of NaOH being applied (0.1 to 1 M), while maintaining the volumes and concentrations of gold precursor and reductant (500 μL, 0.2 mM HAuCl_4_ and 500 μL, 10 mM D-glucose). The initial pH of 0.2 mM gold salt solutions is around 4.3, with the addition of NaOH leading to its increase to values from 9.9 (0.1 M NaOH) to 12.4 (1 M NaOH), as shown in Table S3.

As it can be noted, the optical behavior of the produced AuNPs is one of blue shift of λ_max_ towards lower wavelengths for higher pH, meaning that for the same concentration of glucose, we can achieve size distributions with lower diameters (Figure S1E). This is because hydrolysis of gold salt complex ions happens faster, leading to a higher availability of these ions to be reduced and form gold seeds. Thus, the dynamic range of size variation of particles can be manipulated by applying different NaOH concentrations for hydrolysis. Also, in terms of the kinetic features of the synthesis, pH has an effect of speeding up the nucleation and growth of particles, as showed in Figure S1F and in Table S3, showing the increase in kinetic rate constants value associated with higher pH. Consequently, NaOH can be used to manipulate the colorimetric behavior of the assay, to achieve color variation of particles in the relevant glucose concentration range.

#### 3.1.2. Effect of Reducing Agent Concentration and Direct Colorimetric Glucose Sensing

**Figure 1:**
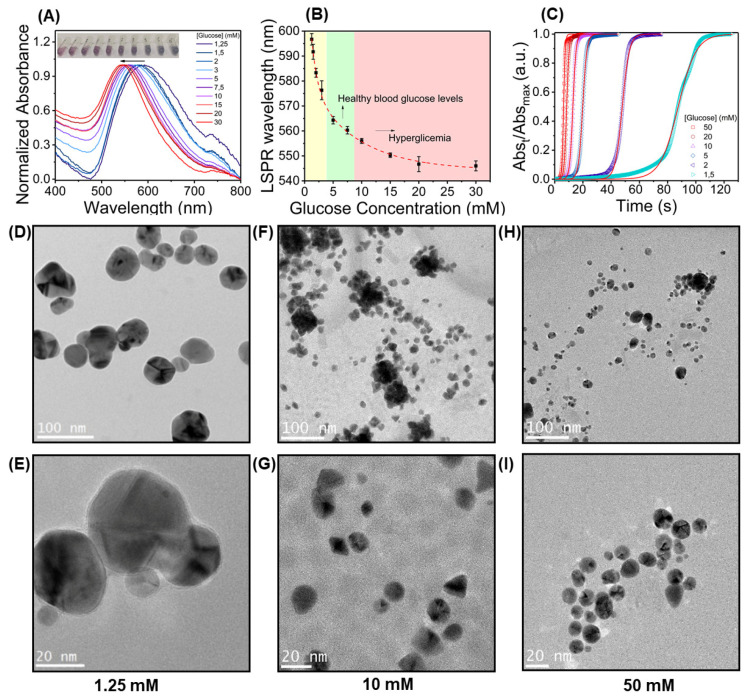
Colorimetric sensing in glucose solutions. (**A**) Colloidal suspensions of gold nanoparticles (AuNPs) produced using different glucose concentrations and corresponding optical spectra. Suspensions have a color evolution from blue (low glucose concentration to red (high glucose concentrations), related to the blue-shift in λ_max_; (**B**) plot of λ_max_ vs. glucose concentration for semi-quantitative sensing approach, showing the decrease in λ_max_ with variation in assay sensitivity (n = 3, r^2^ = 0.996). (**C**) Kinetic curves with Finke–Watzky model fitting for nucleation and growth, showing that the time for the start of AuNPs growth is prolonged when lower glucose concentrations are applied. TEM data for AuNPs formed at different glucose concentrations. (**D**,**E**) 1.25 mM; (**F**,**G**) 10 mM; (**H**,**I**) 50 mM.

Both visual inspection and UV-Vis spectra analysis of colloidal suspensions produced using different glucose concentrations showed evident and marked changes. It is observable by visual inspection that at lower glucose concentrations, the solutions revealed a blue/purple color, whereas at higher glucose concentrations, the solutions became pink-colored (Figure 1A). This is associated with a blue shift of λ_max_ to lower wavelengths and smaller particle size, as glucose concentration increased. λ_max_ fluctuates approximately between 540 nm (at a high glucose concentration of 50 mM) and 600 nm (at a low glucose concentration of 1.25 mM). When plotting the shift in plasmon resonance vs. glucose concentration (Figure 1B), it was verified that the largest LSPR shifts were observed at lower glucose concentrations (below 10 mM). Moreover, changes in plasmon resonance were practically non-existent above 20 mM, revealing a ‘saturation’ behavior at higher glucose concentrations, when available surface area of the synthesized AuNPs able to interact and to be capped by glucose is fully covered. Similar results were achieved when monitoring absorbance intensity for correlation with glucose concentrations, as shown in Figure S3, where the absorbance at 500 and 650 nm was taken for correlation, showing the same alterations in assay sensitivity for higher concentrations. Furthermore, we found that glucose concentration also influences the kinetic features in the formation of the colloidal systems. The less glucose is used in the synthesis, the longer the time delay until the start of AuNPs growth and lower the rate of growth, a similar behavior to the effect of using lower gold salt concentrations, higher volume, and lower NaOH concentrations (Figure 1C and Table S3).

Information regarding the morphology and shape of the AuNPs in colloidal suspensions was collected by TEM. Images of AuNPs synthesized using three different glucose concentrations were obtained: Low concentration (1.25 mM) (Figure 1D,E), intermediate concentration (10 mM) (Figure 1F,G), and high concentration (50 mM) (Figure 1H,I). Firstly, we found that AuNPs have an increasingly symmetrical, regular, and homogeneous morphology when synthesized with high glucose concentrations. In addition, it was also found that nanoparticle size decreases as glucose concentration increases. In fact, AuNPs formed using lower glucose concentrations are significantly larger (50–100 nm of diameter) than those formed using higher glucose concentrations (3–20 nm of diameter). The large nanoparticles formed at a low glucose concentration are apparently the result of a kinetic aggregation mechanism, since the amount of glucose involved in the synthesis is insufficient to cap the entire surface of AuNPs, promoting their aggregation. On the other hand, high amounts of glucose, able to completely cap the nanoparticles surface, hinder their aggregation, resulting in smaller AuNPs. It is also noticeable that the size increase is greater when comparing AuNPs produced with low and intermediate glucose concentrations, as opposed to comparing intermediate to high glucose concentration conditions, which agrees with the decrease in assay sensitivity. Thus, the size difference between the AuNPs at different glucose concentrations correlates nicely with the observed changes in LSPR. Furthermore, it is also noticeable that for higher glucose concentrations, larger sized particles (~50 nm) are formed, whereas for lower glucose concentrations, no smaller particles appear to be synthesized. This shows that size dispersity for AuNPs synthesized with lower glucose concentrations is higher and may affect the optical profiles of colloidal solutions. With further parameter control (e.g., higher temperature, different gold salt, and NaOH concentrations), very uniform particles with thinner resonance band thickness could be achieved.

### 3.2. Colorimetric Assay in Paper Substrate

The translation of the assay into the paper substrate was accomplished through an in-situ synthesis strategy of the glucose-capped particles at the paper surface. The synthesis was performed by subsequent drop-casting of each precursor reagent in a specific order, to meet the requirements of a point-of-care platform, where the sample being analyzed is lastly introduced (Scheme S2). Firstly, gold salt precursor is loaded into the reaction zone and left to dry. Secondly, NaOH is introduced, causing a color change (yellow to grey), due to hydrolysis at paper surface. By adding a drop of glucose standard solution with increasing molar concentrations into each reaction zone, a pink color appears on the paper after a few seconds, corresponding to the synthesis of AuNPs, and the intensity of the developed color increases with the concentration of glucose in solution, as presented in Figure S4. Knowing how each precursor influences the dynamic range of colorimetric behavior, as shown in the previous section, parameter optimization was performed, by increasing the concentrations of precursors drop-casted into the substrate, to attain color signal uniformity, intensity, and stability, with a variation range that allows for discrimination of different glucose concentrations in the physiological range.

#### Parameter Optimization

**Figure 2:**
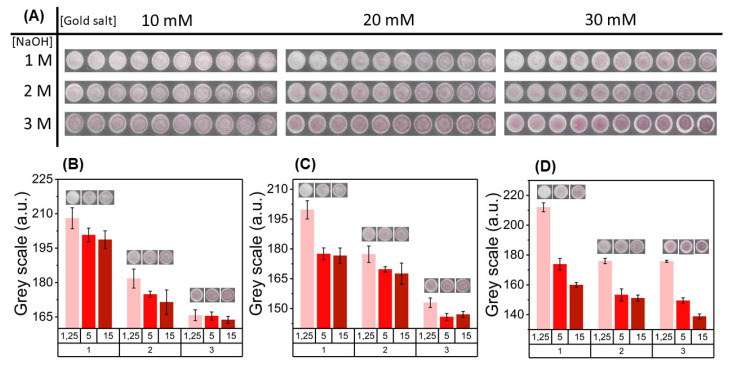
Optimization of colorimetric signals for the paper-based glucose assay. (**A**) Assay performed for different reaction parameters and the corresponding color signals. Three different HAuCl_4_ concentrations (10, 20, and 30 mM, 2.5 μL) and NaOH (1, 2, and 3 M, 2 μL) were used to determine how these reaction parameters influence the color achieved in the reaction zones. (**B**) Histograms of color signal intensity (grey scale value) comparison for 10 mM HAuCl_4_ concentration and three glucose concentrations (1.25, 5, and 15 mM). (**C**) Histograms of color signal intensity (grey scale value) comparison for 20 mM HAuCl_4_ concentration. (**D**) Histograms of color signal intensity (grey scale value) comparison for 30 mM HAuCl_4_ concentration.

As previously mentioned, the colorimetric behavior of the in-situ synthesis at cellulose surface is one of increase in the color intensity. Thus, digital analysis of color signals was carried, firstly using the RGB color space, more specifically, by monitoring the grey scale values of pixels, as they give a measure of how similar the color signal is to the white background of paper. To obtain stronger colorimetric signals, the concentration of gold salt had to be increased in two orders of magnitude (from 0.2 to 10, 20, and 30 mM), as the opacity of the white background does not allow for visual detection of particles synthesized with low concentrations of gold salt. Different combinations of gold salt and NaOH concentrations were tested and are presented in Figure 2. By visual inspection of the results in Figure 2A, it can be noted that both the concentration of gold salt and NaOH have influence over the colorimetric output of the assay. Generally, the increase of gold salt concentration leads to an increase of the color intensity, as well has some alteration in hue. Variation of NaOH concentration introduced in the paper substrate appears to influence the range of colorimetric output of particles. As it can be noted, in some cases, low glucose concentrations are not able to synthesize AuNPs (conditions 20-1 and 30-1 of [HAuCl_4_] and [NaOH]), as the amount of NaOH introduced does not allow for the formation of visible color signals. In contrary, for some conditions (10-3 and 20-3), there seems to be NaOH in excess, as the difference between colorimetric signals for different glucose concentration conditions is smaller and there is not enough sensitivity. Consequently, the range of colorimetric signal variation can be manipulated by changing HAuCl_4_ and NaOH concentration applied to the platform and, thus, meet the physiological range of measurement for glucose. From the different conditions presented in Figure 2B–D and Figure S5, the one using 30 mM of HAuCl_4_ and 3 M of NaOH was selected, as it presents intense and uniform signal, with a suitable range of variation for color signals allowing for a clear distinction between glucose concentration levels.

### 3.3. Color Feature Study and Calibration

**Figure 3:**
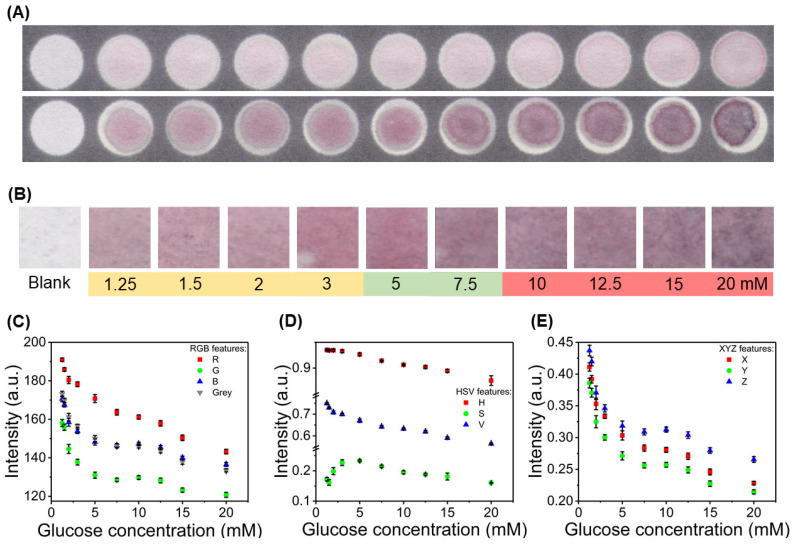
Color signal for the optimized assay condition (30 mM HAuCl_4_ and 3 M NaOH) and digital color analysis of Red, Green, Blue (RGB), Hue, Saturation, and Value (HSV), and XYZ color space features. (**A**) Comparison between color signal for the optimized condition and an alternative condition with an appropriate color range variation for glucose measuring, showing marked differences in color intensity and hue. (**B**) Crop sections extracted from the reaction zones using a custom image processing software, showing the variation of the color signal with increasing glucose concentrations. (**C**) Analysis of RGB color space features correlated with glucose concentration. (**D**) Analysis of HSV color space features correlated with glucose concentration. (**E**) Analysis of XYZ color space features correlated with glucose concentration (n = 6).

Taking the chosen optimized condition for the paper-based assay, analysis of the colorimetric signals for different glucose concentrations was performed. Features from the RGB, HSV, and XYZ color spaces were analyzed, to obtain correlations between them and the applied glucose concentration, to achieve a calibration for a semi-quantitative sensing approach. The colorimetric signals and the plot of features vs. glucose concentration are presented in Figure 3. In Figure 3A, the optimized condition is compared with another that shows an appropriate range of color variation for glucose quantification (10 mM HAuCl_4_; 1 m NaOH). It can be noted that there is a much more intense colorimetric output and a detectable variation in hue. An example of the extracted crop sections from each of the reaction zones is presented in Figure 3B. It can be noted that the color for low glucose concentrations has a pink/pale red hue, which gets more intense with the increase of glucose quantity. Between 5 and 7.5 mM of glucose, there is a slight change of hue to purple, that also gets more intense as the concentrations are further increased. This way, there is the possibility for qualitative detection of glucose with the naked eye, because for glucose concentration above the healthy range, there is a characteristic purple hue.

The mean values of features for each crop were computed and correlated to the corresponding glucose concentration, as can be seen in Figure 3C–E. For the features in the RGB color space, there is a general decrease in their intensity, as the color signal gets more intense and further in value when compared to the white background. Regarding the HSV color space, the features behave differently. For Hue and Value, there is a decrease associated with the increase of glucose concentration. However, for the Saturation feature, there is an initial increase until 5 mM, followed by a decrease in its intensity. For XYZ color space, the behavior is similar to the RGB color space, as it consists in a matrix transformation of the RGB space. Furthermore, color signal stability was studied and is presented in Figure S6, showing that the difference in color intensity between the lower (1.25 mM) and higher (20 mM) glucose concentration conditions decreases with time. This is due to the evaporation of the aqueous sample matrix, following the introduction of the sample into the reaction zone. Taking these results into account, the color signals were taken by scanning of the paper platform 2 min after assay performance, as the difference between color intensity is in its maximum.

**Figure 4:**
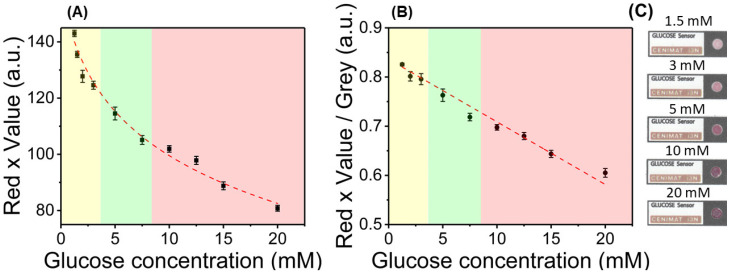
Calibration curves for the colorimetric, non-enzymatic paper-based assay. (**A**) Calibration curve using the combination of the Red and Value channels from RGB and HSV color spaces (*y* = 163.89 − 26.65 ln(*x* + 1.20), *r*^2^ = 0.9886, n = 6). (**B**) Linear calibration curve obtained by the ratio between the previous feature combination (R × V) and the grey scale value (*y* = 0.836 − 0.013*x*, *r*^2^ = 0.982, n = 6). (**C**) Paper-based platform for assay performance, showing the color signals for different glucose concentrations.

From these results, a selection of features was made to achieve calibration curves, with plots presented in Figure 4. Taking the Red feature from the RGB color space and Value feature of the HSV color space, a calibration for semi-quantitative glucose sensing was obtained (Red × Value), showing a negative logarithmic dependency towards glucose concentration. The detection limit of the assay using this metric for calibration is of 0.44 mM. In addition, a linear calibration curve was obtained, computing the ratio between the presented metric and the grey scale intensity (Red × Value/Grey), with a detection limit of 0.65 mM and sensitivity of 0.013 AU/mM. Both calibration curves can be used individually or simultaneously, to quantify glucose in the physiological range, with an appropriate sensitivity to distinguish different states of blood sugar (hypoglycemia, healthy levels, and hyperglycemia). However, a linear calibration until 20 mM is more suitable for diabetes management, as more control can be achieved for hyperglycemic states. Consequently, the (Red × Value/Grey) ratio would be the preferred metric for correlation with glucose levels.

### 3.4. Interference Assay in the Paper Platform

**Figure 5:**
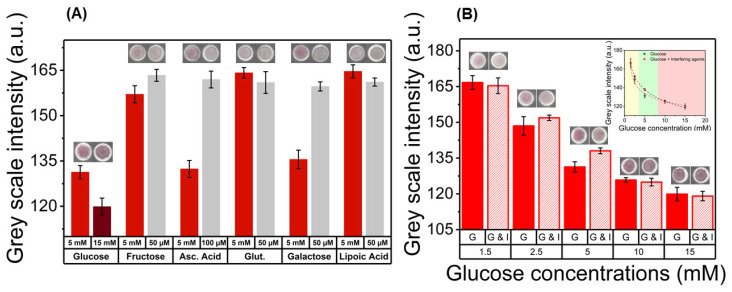
Interference assay for assessment of specificity towards glucose. (**A**) Color signals produced using glucose and common reducing agents individually. (**B**) Color signals obtained for standard samples containing glucose and interfering agents (G & I), with concentration in the physiological levels, are compared with signals containing glucose individually (G) showing the effect of these interfering agents. The plot of correlation between grey scale value and glucose concentrations are presented in inset.

An interference assay was performed, with the purpose of evaluating if common reducing molecules present in physiologic samples, other than glucose (fructose, ascorbic acid, glutathione, galactose, and lipoic acid), have the capability of synthesizing AuNPs in the paper substrate and influence the color signals. Results are presented in Figure 5 and Tables S4 and S5. We found that when the same concentrations of glucose and interfering agents (5 mM) are applied individually, some of the reducing agents can produce AuNPs. In the case of fructose, ascorbic acid, and galactose, color appears in the reaction zones, as showed by the grey scale values (Figure 5A), showing that these molecules have enough reducing potential to promote in-situ synthesis of AuNPs. However, this value of concentration is close to 100-fold more concentrated then the normal levels of these molecules in blood samples (in the μM range). Consequently, lower concentrations of the reducing agents, at physiological levels, were tested (50 μM for fructose, glutathione, galactose, and lipoic acid; 100 μM for ascorbic acid). In this case, we found that there is no appearance of the red/pink color signal characteristic of the formation of AuNPs in paper, showing that these molecules are silent and alone, and are not able to synthesize AuNPs. This results in a good specificity of the sensing mechanism, since in physiologically relevant concentrations, potential interferents do not have sufficient reducing potential to promote AuNPs synthesis.

For samples containing different glucose concentrations, simultaneously with the interfering agents, the colorimetric behavior of the assay is maintained. As it can be seen in Figure 5B, the grey scale intensity shows its characteristic decrease, that is maintained when the interfering agents are present. Furthermore, the presence of interfering agents does not greatly influence the grey scale values for most of the tested concentrations. Their presence only seems to slightly decrease the color intensity for glucose concentrations bellow 10 mM. Similar results were achieved for the Red × Value metric, presented in Figure S7. This translates into an improvement in assay sensitivity, as the changes in the grey scale intensity for concentrations in the hyperglycemic interval show a greater difference when compared to the ones in healthy blood sugar interval (Figure 5B and Figure S6—inset). Consequently, we found that this colorimetric, paper-based enzyme-free assay presents a suitable specificity towards glucose, as the presence of interfering agents does not lead to significant changes in the color signals and does not hinder the capability to distinguish glucose concentrations both qualitatively, by visual inspection, and semi-quantitatively, through digital colorimetric analysis.

### 3.5. AuNPs Characterization in Paper Substrate

**Figure 6:**
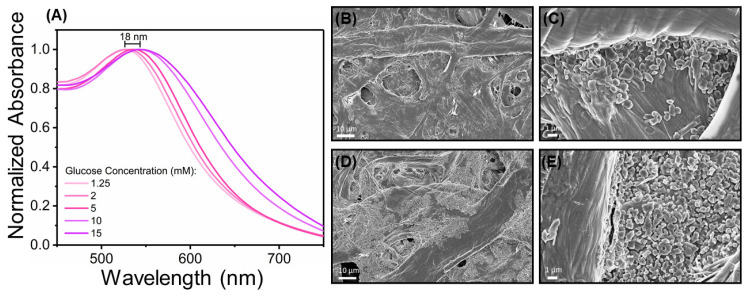
Optical and morphological characterization of in-situ synthesized AuNPs. (**A**) Absorbance measurements of paper substrate with AuNPs synthesized using different glucose concentrations, showing a red-shift associated with glucose increase. (**B**,**C**) SEM images of AuNPs synthesized using 2 mM glucose concentration. (**D**,**E**) SEM images of AuNPs synthesized using 15 mM glucose concentration.

Characterization of in-situ synthesized AuNPs promoted by glucose was performed, to better understand the mechanisms underlying the produced color signals and the optical and morphological characteristics of AuNPs. UV-Vis spectrophotometric measures of paper substrates with produced AuNPs was performed, to identify the plasmonic characteristics of AuNPs and their absorbance profiles. Results showed that the characteristic LSPR absorbance spectra could be tracked, with variations in hue being accompanied by a red-shift of the absorbance spectra towards higher wavelengths, which is in accordance with the color achieved in the paper substrate (Figure 6A). For color signals associated with low glucose concentrations (pink hue), the peak absorbance wavelength presents lower values, around 526 nm, getting higher when increasing concentrations of glucose are present (purple hue), around 544 nm, with a red-shift of 18 nm. When compared to AuNPs synthesized in aqueous medium, the spectral behavior is the opposite, as in this colloidal gold suspensions, there is a blue-shift associated with increase in glucose concentration. This is apparently due to the reduction reaction occurring in a more localized fashion, at cellulose surface. To track structural changes in the synthesized AuNPs and correlate them to the spectral behavior, SEM was performed on AuNPs synthesized using two different glucose concentrations (Figure 6B–E). Results showed that particles in the tens to hundreds of nanometers are formed. For low glucose concentration (2 mM, Figure 6B,C), less particles are present at cellulose surface, while for higher concentration (15 mM, Figure 6D,E), large clusters of particles appear at fibers’ surface, at a much higher density. Thus, the changes in hue and the shift in absorbance is apparently caused by a higher number of particles being formed. As more glucose is present, it consumes more complex gold ions that were in excess and not fully consumed when lower glucose concentrations are applied, leading to the formation of greater amounts of particles in large clusters. This leads to the variation of plasmonic properties of the particles as a bulk, thus leading to the change in hue. When compared to the synthesis of AuNPs in solution, it is apparent that nucleation and growth properties of AuNPs are different. In solution, gold atoms undergo Brownian motion, being readily available for the formation and growth of nanoparticle seeds. However, this is not the case in the paper substrate, as gold complex ions are deposited at cellulose surface and not in motion, leading to a different range of particle sizes for different glucose concentrations, thus presenting a characteristic pink/red color, as presented in Figure 2 and Figure 3.

The presence of the synthesized AuNPs on the paper was also proved by X-Ray Diffraction (XRD) (Figure S8A). The addition of HAuCl_4_ and NaOH to the Whatman paper does not correspond to the formation of any crystallographic structure, since all detected peaks are assigned to the crystallographic structure of type I cellulose. However, after adding glucose, another peak was observed around 38.5°, which is assigned to the crystallographic structure of gold, corresponding to the crystal plane with Miller index (111). Lastly, SERS was also used to test these AuNPs on paper as a potential SERS platform. The signal of a Raman spectrum compared to a representative SERS spectrum of R6G is shown in Figure S8B,C. In this case, an enhancement of the Raman signal corresponding to R6G on paper with AuNPs was observed, when compared to the signal obtained for R6G on glass. It was possible to identify in both spectra two characteristic lines at 1360 and 1510 cm^−1^, corresponding to the Raman vibrations associated with R6G. These areas were used to calculate spectral intensity and, consequently, the respective EF. An average EF value of (4.6 × 10^4^) for the 1360 cm^−1^ line and an average EF value of (1.6 × 10^4^) for the 1510 cm^−1^ line were obtained, corresponding to a considerable enhancement of Raman signal due to SERS effect.

### 3.6. Colorimetric Sensing in Biological Samples

The application of this sensing assay for biological samples was performed using human serum. Because this protocol is based on the formation of AuNPs through the direct reduction of gold salt by glucose, the matrix effect was considered, and an appropriate sample treatment was applied. Proteins are known to interact with AuNPs through the formation of a protein corona around particle’s surface. [48] Therefore, a protein precipitation protocol was used to remove as much proteins as possible, as is commonly done in nanoparticle based colorimetric assays [49], because their presence hinders the capability of formation, growth and aggregation of particles.

**Figure 7:**
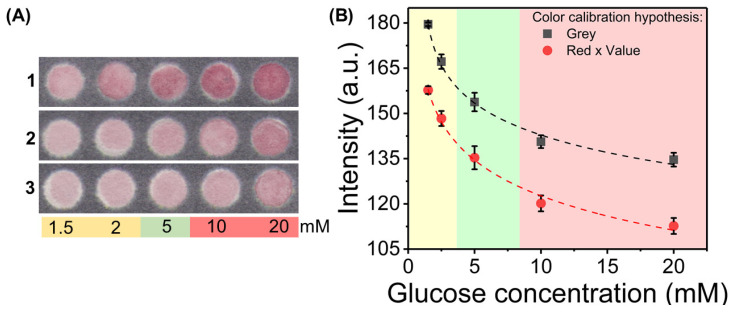
Assay performed with serum samples. (**A**) Color signals obtained for differently fold diluted serum samples (1–25%; 2–33%; 3–50%), with various spiked glucose concentrations. The color signals show a decrease of color intensity with the increase of serum concentration, due to the lower efficacy of protein removal. (**B**) Color signal calibration for 25% diluted serum samples (n = 5), showing that these complex samples lead to the same behavior of color signals, able to differentiate different blood glucose states, in a semi-quantitative approach.

The colorimetric results are presented in Figure 7. As it can be seen, the synthesis was successfully replicated using serum samples, showing the same color evolution with the increase in spiked glucose concentrations (increase in color intensity). However, there is an effect of the degree of protein precipitation towards the intensity of the color signals. For a serum:ethanol ratio of 1:3 (25% serum), there are more proteins being precipitated, thus reducing the hindering effect of proteins towards the nucleation and growth of particles in the paper substrate (Figure 7A(1)). When lower amounts of ethanol are used, protein precipitation is not so efficient, thus higher concentration of proteins remain in the sample. This leads to a decrease in the resulting color intensity when spiked samples are introduced (Figure 7A(2,3)). To better understand this mechanism, a colorimetric protein assay using the Biuret reagent was performed (Figure S9), with results showing that for a 1:3 serum to ethanol ratio, there is approximately 1000-fold reduction in protein concentration in serum samples, equivalent to 0.1% serum sample.

Correlating color signals obtained using 25% serum samples with glucose concentrations, we found that the same behavior for the grey scale intensity and Red x Value metric is achieved (Figure 7B), with a decrease in their values in a negative logarithmic dependency towards glucose concentrations. This shows that with proper sample treatment for protein precipitation and removal, which can be integrated in a paper-based platform, a colorimetric, enzyme-free, semi-quantitative sensing approach can be achieved for glucose sensing. A critical comparison between the analytical performance of this paper-based platform with others recently presented in literature is presented in Table 1.

## 4. Conclusions

Easy access to simple, reliable, and effective diagnostic methods is one of the most important aspects in prevention and treatment of diseases. Although these diagnostic tools are currently quite common and generally available in developed countries, their need is greatest mostly in economically and socially underprivileged regions, where the access to medical infrastructures is limited and not specialized. Therefore, work has been carried out to provide cheap, rapid, yet robust and portable molecular diagnostic platforms for use at peripheral laboratories and point-of-care testing. These diagnostic platforms are particularly relevant and useful for diseases with high prevalence worldwide, as in the case of diabetes mellitus. So, in this work, we developed a novel, inexpensive, rapid, disposable, enzyme-free and colorimetric paper-based platform for glucose sensing in the physiological range, with potential for use in the point-of-care. Contrary to the typical glucose detection tests, this method is enzyme-free and based on the principle that glucose can act as both a reducing and capping agent for AuNPs synthesis, whose size corresponds to a color shift depending on the glucose concentration. AuNPs formation provided by the reducing action of glucose is reflected in the appearance of a red color on the paper substrate, which becomes more intense and varies its hue as glucose concentration increases. The developed platform was tested and calibrated using standard solutions with increasing molar concentrations of glucose (1.25–20 mM), resulting in reliable calibration curves using two metrics (Red × Value and Red × Value/Grey). The colorimetric results acquired with a commercial scanner and analyzed by digital image processing resulted in appropriate ranges of variation for the measurement of glucose in the physiological range, with good sensitivity and specificity, in a cheap, efficient way, with reliable results within 2 min of assay performance. A first validation of this paper-based method was performed with human serum samples, showing that the direct synthesis of AuNPs using complex solutions is successful. Using spiked serum samples treated to remove the matrix effect through a protein precipitation method, the applicability of this assay was proved, as the behavior of increased color intensity is achieved, and glucose concentrations can be discerned. This pre-treatment method showed efficacy for decreasing protein influence in AuNPs synthesis, but other important aspects such as the reducing potential of certain proteins towards the formation of particles should be investigated, so that in-situ nanomaterial synthesis-based assays find wider applications. With this work, enzyme-free colorimetric sensing of glucose in paper platforms is described for the first time, as an alternative for conventional electrochemical sensing, with the developed platform being easy to use, without the need for expensive and complex laboratory set ups, representing a simpler, faster, and low-cost alternative with potential to be applied to the point-of-care. With further development of such colorimetric, non-enzymatic methods, sampling and sample processing in paper substrate, fully functional paper-based analytical devices can be developed and combined with digital, smartphone-based tools for automatic color signal analysis, resulting in systems that can compete with existing glucose detection systems, with elevated sustainability.

## Figures and Tables

**Table 1:** Comparison of analytical performance of paper-based colorimetric platforms for glucose level determination.

Type	Platform Material	Linear Range	Detection Limit	Reference
Enzymatic—GOx/HRP/4-aminoantipyrine	Chromatography paper	0.5–10 mM	0.9 mM	[50]
Enzymatic—GOx/HRP/3 different chromogenic indicators	Chromatography paper	1.0–6.0 mM	12 µM	[51]
Enzymatic—GOx/HRP/ABTS	Carboxyl nanocellulose	1.5–13 mM	1.5 mM	[52]
Enzymatic—GOD/HRP byenzyme system	Chromatography paper	1.0–11 mM	0.3 mM	[53]
Enzymatic—GOx/HRP/TMB	Chromatography paper	0.1–1.0 mM	50 µM	[54]
Non-enzymatic—AuNPs in-situ synthesis	Chromatography paper	1.25–20 mM	0.65 mM	This work

## Supplementary Materials

The following are available online at https://www.mdpi.com/2079-4991/10/10/2027/s1, Scheme S1: Schematic of Lab-on-paper technology used in paper-based platform manufacturing. Scheme S2: Schematic of colorimetric, non-enzymatic paper-based assay steps for glucose sensing. Tables S1–S3: Effect of precursor reagents towards optical and kinetic properties of AuNPs synthesis. Tables S4 and S5: Interference assay in paper substrate. Figure S1: Effect of reaction parameters towards optical and kinetic properties. Figure S2: Curves for derivative of experimental Abs_t_ data. Figure S3: Correlation of normalized absorbance at 500 and 650 nm towards glucose concentrations. Figure S4: Glucose detection approach in paper substrate. Figure S5: Optimization of colorimetric signals for Red × Value metric. Figure S6: Color signal stability analysis. Figure S7: Interference assay results for the Red × Value metric. Figure S8: Characterization of AuNPs synthesized on paper. Figure S9: Study of protein precipitation and removal efficacy using ethanol.

## References

[1] Costa M.N., Veigas B., Jacob J.M., Santos D.S., Gomes J., Baptista P.V., Martins R., Inácio J., Fortunato E. (2014). A Low Cost, Safe, Disposable, Rapid and Self-Sustainable Paper-Based Platform for Diagnostic Testing: Lab-on-Paper. Nanotechnology.

[2] Chen X., Chen J., Wang F., Xiang X., Luo M., Ji X., He Z. (2012). Determination of Glucose and Uric Acid with Bienzyme Colorimetry on Microfluidic Paper-Based Analysis Devices. Biosens. Bioelectron..

[3] Liu S., Su W., Ding X. (2016). A Review on Microfluidic Paper-Based Analytical Devices for Glucose Detection. Sensors.

[4] Vaurs C., Brun J.F., Bertrand M., Burcelin R., Du Rieu M.C., Anduze Y., Hanaire H., Ritz P. (2016). Post-Prandial Hypoglycemia Results from a Non-Glucose-Dependent Inappropriate Insulin Secretion in Roux-En-Y Gastric Bypassed Patients. Metabolism.

[5] Vashist S. (2013). Continuous Glucose Monitoring Systems: A Review. Diagnostics.

[6] American Diabetes Association (2010). Diagnosis and Classification of Diabetes Mellitus. Diabetes Care.

[7] Aronson D. (2008). Hyperglycemia and the Pathobiology of Diabetic Complications. Cardiovascular Diabetology: Clinical, Metabolic and Inflammatory Facets.

[8] Wang H.C., Lee A.R. (2015). Recent Developments in Blood Glucose Sensors. J. Food Drug Anal..

[9] Chen C., Zhao X.-L., Li Z.-H., Zhu Z.-G., Qian S.-H., Flewitt A. (2017). Current and Emerging Technology for Continuous Glucose Monitoring. Sensors.

[10] Wang J. (2008). Electrochemical Glucose Biosensors. Chemical Reviews.

[11] Heller A., Feldman B. (2008). Electrochemical Glucose Sensors and Their Applications in Diabetes Management. Chemical Reviews.

[12] Martinez A.W., Phillips S.T., Whitesides G.M., Carrilho E. (2010). Diagnostics for the Developing World: Microfluidic Paper-Based Analytical Devices. Anal. Chem..

[13] Martinez A.W., Phillips S.T., Butte M.J., Whitesides G.M. (2007). Patterned Paper as a Platform for Inexpensive, Low-Volume, Portable Bioassays. Angew. Chem. Int. Ed..

[14] Marques A.C., Santos L., Costa M.N., Dantas J.M., Duarte P., Gonçalves A., Martins R., Salgueiro C.A., Fortunato E. (2015). Office Paper Platform for Bioelectrochromic Detection of Electrochemically Active Bacteria Using Tungsten Trioxide Nanoprobes. Sci. Rep..

[15] Si P., Huang Y., Wang T., Ma J. (2013). Nanomaterials for Electrochemical Non-Enzymatic Glucose Biosensors. RSC Adv..

[16] Revathi C., Rajendra Kumar R.T. (2019). Enzymatic and Nonenzymatic Electrochemical Biosensors. Fundamentals and Sensing Applications of 2D Materials.

[17] Qin L., Zeng G., Lai C., Huang D., Xu P., Zhang C., Cheng M., Liu X., Liu S., Li B. (2018). “Gold Rush” in Modern Science: Fabrication Strategies and Typical Advanced Applications of Gold Nanoparticles in Sensing. Coordination Chemistry Reviews.

[18] Zhang Z., Wang H., Chen Z., Wang X., Choo J., Chen L. (2018). Plasmonic Colorimetric Sensors Based on Etching and Growth of Noble Metal Nanoparticles: Strategies and Applications. Biosensors and Bioelectronics.

[19] Nasir M., Nawaz M.H., Latif U., Yaqub M., Hayat A., Rahim A. (2017). An Overview on Enzyme-Mimicking Nanomaterials for Use in Electrochemical and Optical Assays. Microchimica Acta.

[20] Hwang D.W., Lee S., Seo M., Chung T.D. (2018). Recent Advances in Electrochemical Non-Enzymatic Glucose Sensors—A Review. Analytica Chimica Acta.

[21] Guo Y., Zhao W. (2019). In Situ Formed Nanomaterials for Colorimetric and Fluorescent Sensing. Coordination Chemistry Reviews.

[22] Li T., Zhu K., He S., Xia X., Liu S., Wang Z., Jiang X. (2011). Sensitive Detection of Glucose Based on Gold Nanoparticles Assisted Silver Mirror Reaction. Analyst.

[23] Xia Y., Ye J., Tan K., Wang J., Yang G. (2013). Colorimetric Visualization of Glucose at the Submicromole Level in Serum by a Homogenous Silver Nanoprism-Glucose Oxidase System. Anal. Chem..

[24] Nair P.A., Sreenivasan K. (2016). Non Enzymatic Colorimetric Detection of Glucose Using Cyanophenyl Boronic Acid Included β-Cyclodextrin Stabilized Gold Nanoparticles. Anal. Methods.

[25] Gao Y., Wu Y., Di J. (2017). Colorimetric Detection of Glucose Based on Gold Nanoparticles Coupled with Silver Nanoparticles. Spectrochim. Acta Part A Mol. Biomol. Spectrosc..

[26] Cai T., Gao Y., Yan J., Wu Y., Di J. (2017). Visual Detection of Glucose Using Triangular Silver Nanoplates and Gold Nanoparticles. RSC Adv..

[27] Adnan S., Kalwar N.H., Abbas M.W., Soomro R.A., Saand M.A., Awan F.R., Avci A., Pehlivan E., Bajwa S. (2019). Enzyme-Free Colorimetric Sensing of Glucose Using l-Cysteine Functionalized Silver Nanoparticles. SN Appl. Sci..

[28] Juřík T., Podešva P., Farka Z., Kovář D., Skládal P., Foret F. (2016). Nanostructured Gold Deposited in Gelatin Template Applied for Electrochemical Assay of Glucose in Serum. Electrochim. Acta.

[29] Scandurra A., Ruffino F., Sanzaro S., Grimaldi M.G. (2019). Laser and Thermal Dewetting of Gold Layer onto Graphene Paper for Non-Enzymatic Electrochemical Detection of Glucose and Fructose. Sens. Actuators B Chem..

[30] Buk V., Pemble M.E., Twomey K. (2019). Fabrication and Evaluation of a Carbon Quantum Dot/Gold Nanoparticle Nanohybrid Material Integrated onto Planar Micro Gold Electrodes for Potential Bioelectrochemical Sensing Applications. Electrochim. Acta.

[31] Buk V., Pemble M.E. (2019). A Highly Sensitive Glucose Biosensor Based on a Micro Disk Array Electrode Design Modified with Carbon Quantum Dots and Gold Nanoparticles. Electrochim. Acta.

[32] Branagan D., Breslin C.B. (2019). Electrochemical Detection of Glucose at Physiological PH Using Gold Nanoparticles Deposited on Carbon Nanotubes. Sens. Actuators B Chem..

[33] Carrilho E., Martinez A.W., Whitesides G.M. (2009). Understanding Wax Printing: A Simple Micropatterning Process for Paper-Based Microfluidics. Anal. Chem..

[34] Zhao W., Van Den Berg A. (2008). Lab on Paper. Lab on a Chip.

[35] Renault C., Koehne J., Ricco A.J., Crooks R.M. (2014). Three-Dimensional Wax Patterning of Paper Fluidic Devices. Langmuir.

[36] Unser S., Campbell I., Jana D., Sagle L. (2015). Direct Glucose Sensing in the Physiological Range through Plasmonic Nanoparticle Formation. Analyst.

[37] Raveendran P., Fu J., Wallen S.L. (2003). Completely “Green” Synthesis and Stabilization of Metal Nanoparticles. J. Am. Chem. Soc..

[38] Raveendran P., Fu J., Wallen S.L. (2006). A Simple and “Green” Method for the Synthesis of Au, Ag, and Au–Ag Alloy Nanoparticles. Green Chem..

[39] Psychogios N., Hau D.D., Peng J., Guo A.C., Mandal R., Bouatra S., Sinelnikov I., Krishnamurthy R., Eisner R., Gautam B. (2011). The Human Serum Metabolome. PLoS ONE.

[40] Oliveira M.J., Quaresma P., De Almeida M.P., Araújo A., Pereira E., Fortunato E., Martins R., Franco R., Águas H. (2017). Office Paper Decorated with Silver Nanostars-an Alternative Cost Effective Platform for Trace Analyte Detection by SERS. Sci. Rep..

[41] Engelbrekt C., Sørensen K.H., Zhang J., Welinder A.C., Jensen P.S., Ulstrup J. (2009). Green Synthesis of Gold Nanoparticles with Starch-Glucose and Application in Bioelectrochemistry. J. Mater. Chem..

[42] Suvarna S., Das U., Sunil K.C., Mishra S., Sudarshan M., Saha K.D., Dey S., Chakraborty A., Narayana Y. (2017). Synthesis of a Novel Glucose Capped Gold Nanoparticle as a Better Theranostic Candidate. PLoS ONE.

[43] Corma A., Garcia H. (2008). Supported Gold Nanoparticles as Catalysts for Organic Reactions. Chemical Society Reviews.

[44] Ivanova S., Petit C., Pitchon V. (2004). A New Preparation Method for the Formation of Gold Nanoparticles on an Oxide Support. Appl. Catal. A Gen..

[45] Pacławski K., Streszewski B., Jaworski W., Luty-Błocho M., Fitzner K. (2012). Gold Nanoparticles Formation via Gold(III) Chloride Complex Ions Reduction with Glucose in the Batch and in the Flow Microreactor Systems. Colloids Surf. A Physicochem. Eng. Asp..

[46] Watzky M.A., Finke R.G. (1997). Transition Metal Nanocluster Formation Kinetic and Mechanistic Studies. A New Mechanism When Hydrogen Is the Reductant: Slow, Continuous Nucleation and Fast Autocatalytic Surface Growth. J. Am. Chem. Soc..

[47] Luty-Błocho M., Wojnicki M., Fitzner K. (2017). Gold Nanoparticles Formation via Au(III) Complex Ions Reduction with l-Ascorbic Acid. Int. J. Chem. Kinet..

[48] Charbgoo F., Nejabat M., Abnous K., Soltani F., Taghdisi S.M., Alibolandi M., Thomas Shier W., Steele T.W.J., Ramezani M. (2018). Gold Nanoparticle Should Understand Protein Corona for Being a Clinical Nanomaterial. J. Control. Release.

[49] Huang P.C., Gao N., Li J.F., Wu F.Y. (2018). Colorimetric Detection of Methionine Based on Anti-Aggregation of Gold Nanoparticles in the Presence of Melamine. Sens. Actuators B Chem..

[50] Aksorn J., Teepoo S. (2020). Development of the Simultaneous Colorimetric Enzymatic Detection of Sucrose, Fructose and Glucose Using a Microfluidic Paper-Based Analytical Device. Talanta.

[51] Gabriel E.F.M., Garcia P.T., Cardoso T.M.G., Lopes F.M., Martins F.T., Coltro W.K.T. (2016). Highly Sensitive Colorimetric Detection of Glucose and Uric Acid in Biological Fluids Using Chitosan-Modified Paper Microfluidic Devices. Analyst.

[52] Neubauerova K., Carneiro M.C.C.G., Rodrigues L.R., Moreira F.T.C., Sales M.G.F. (2020). Nanocellulose- Based Biosensor for Colorimetric Detection of Glucose. Sens. Bio Sens. Res..

[53] Zhu W.J., Feng D.Q., Chen M., Chen Z.D., Zhu R., Fang H.L., Wang W. (2014). Bienzyme Colorimetric Detection of Glucose with Self-Calibration Based on Tree-Shaped Paper Strip. Sens. Actuators B Chem..

[54] Gabriel E.F.M., Garcia P.T., Lopes F.M., Coltro W.K.T. (2017). Paper-Based Colorimetric Biosensor for Tear Glucose Measurements. Micromachines.

