# Supplementary material for: Paper-Based In-Situ Gold Nanoparticle Synthesis for Colorimetric, Non-Enzymatic Glucose Level Determination
Source: Nanomaterials (Basel). 2020 Oct 14;10(10):2027. doi: 10.3390/nano10102027 (PMC7602483; doi:10.3390/nano10102027)
Supplement: Supplementary file 1 [file nanomaterials-10-02027-s001.pdf]

Supporting Information

Paper-Based In-Situ Gold Nanoparticle Synthesis for Colorimetric, Non-Enzymatic Glucose Level Determination

Tomás Pinheiro [1], João Ferrão [1], Ana C. Marques [1], Maria J. Oliveira [1], Nitin M. Batra [2], Pedro M. F. J. Costa [2], M. Paula Macedo [3,4], Hugo Águas [1], Rodrigo Martins [1] and Elvira Fortunato [1,*]

[1] CENIMAT/i3N, Materials Science Department, Faculdade de Ciência e Tecnologia–Universidade Nova de Lisboa, Lisbon 2829-516, Portugal; tp.pinheiro@campus.fct.unl.pt (T.P.); j.ferrao@campus.fct.unl.pt (J.F.); accm@campus.fct.unl.pt (A.C.M.); mj.oliveira@campus.fct.unl.pt (M.J.O.); hma@fct.unl.pt (H.Á.); rfpm@fct.unl.pt (R.M.)

[2] Physical Science and Engineering Division, King Abdullah University of Science and Technology (KAUST), Thuwal 23955-6900, Saudi Arabia; nitinkumar.batra@kaust.edu.sa (N.M.B.); pedro.dacosta@kaust.edu.sa (P.M.F.J.C.)

[3] CEDOC, Chronic Disease Research Centre, NOVA Medical School, Faculdade de Ciências Médicas, Universidade NOVA de Lisboa, Campo Mártires da Pátria, Lisbon 1150-190, Portugal; paula.macedo@nms.unl.pt

[4] Education and Research Centre, APDP-Diabetes Portugal (APDP-ERC), Lisbon 1250-203, Portugal

* Correspondence: emf@fct.unl.pt

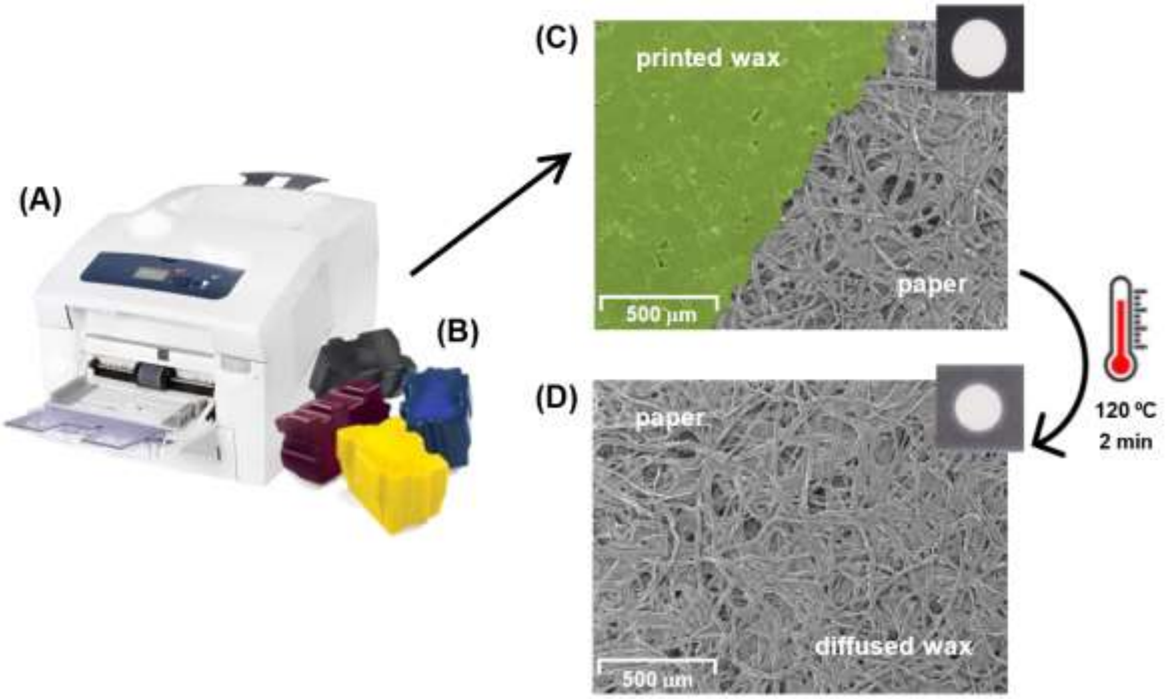

Scheme S1. Schematic of the Lab-on-Paper technology used in the paper-based platforms manufacturing. (**A**) Xerox ColorQube 8570 solid ink printer; (**B**) Solid ink (wax) cartridges; (**C**) SEM image of the surface of Whatman no. 1 paper with (green layer) and without printed wax (before

diffusing); (**D**) SEM image of the paper surface after diffusing the wax through the whole thickness of the paper, creating the hydrophobic barriers that define hydrophilic test zon

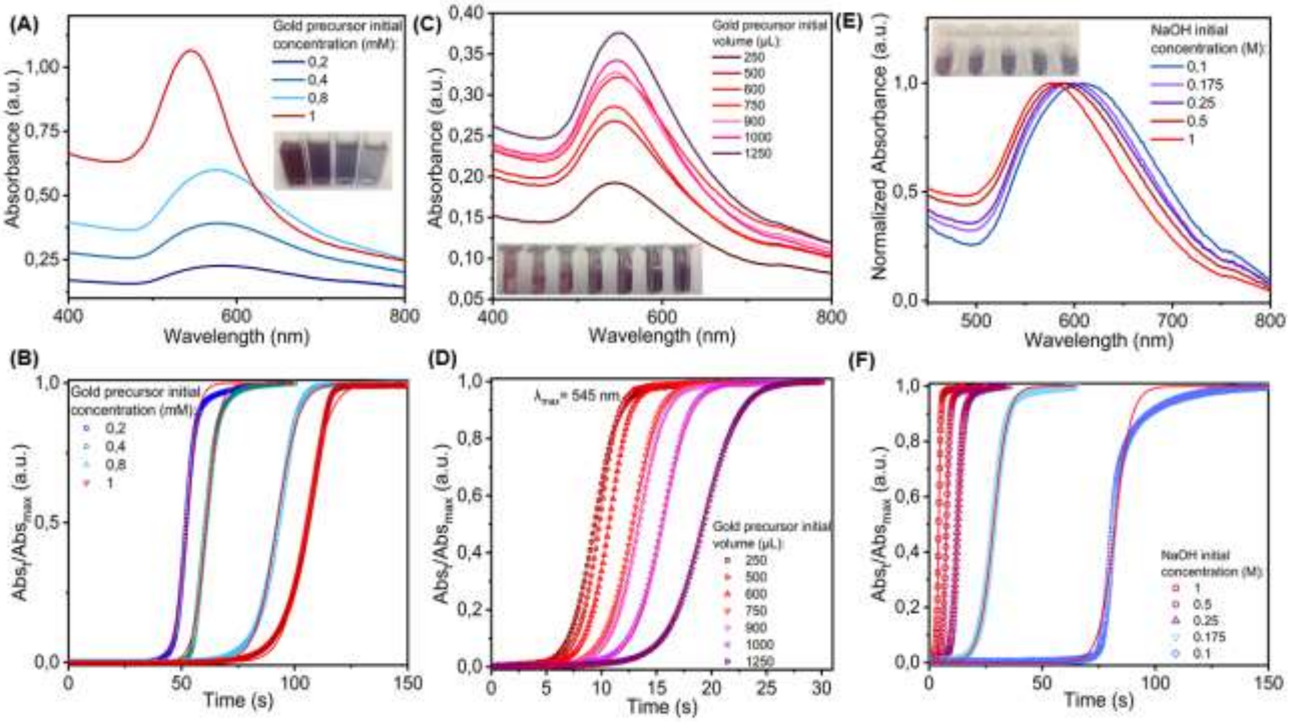

Figure S1. Effect of reaction parameters towards optical and kinetic properties. (A and B) Optical and kinetic curves of colloidal suspension produced using different initial gold salt concentrations. (C and D) Optical and kinetic curves of colloidal suspension produced using different initial gold salt volumes. (E and F) Optical and kinetic curves of colloidal suspension produced using different initial NaOH concentrations.

Through the fitting of experimental Abs_t curves obtained by time-resolved, in-situ UV-Vis spectrophotometric measures for λ_{max}, the kinetic rate constants (k_1—nucleation rate constant; k_2—growth rate constant) were computed and are presented in the following tables, along the corresponding λ_{max}.

The general behavior of the rate constants is as follows: when there is an increase in the time for AuNPs growth to start, the nucleation rate constant (k_1) suffers a general decrease in its values. When the rate of AuNPs growth increases (derivative of experimental rate curves presented in **Figure S2**) and the slope at the steep increase in absorbance is higher, there is a general increase in values for the growth rate constant (k_2).

Table 1. Effect of gold salt initial concentration ($[HAuCl_4]$) towards optical and kinetic properties.

**C_{Au} (M)**	**k_1 (x 10^{-10} s^{-1})**	**k_2 (M^{-1} s^{-1})**	**λ_{max} (nm)**
1	2.40	0.19	584
0.8	7.98	0.21	584
0.4	5.43	0.33	576
0.2	6.27	0.38	547

Table S2. Effect of gold salt initial volume towards kinetic rate constants.

V_{Au} (μL)	k_1 (s^{-1})	k_2 (M^{-1} s^{-1})
250	2.034E-4	0.894
500	3.249E-5	1.065
600	2.449E-5	0.995
750	2.088E-5	0.827
900	1.539E-5	0.813
1000	5.993E-6	0.761
1250	5.301E-6	0.606

Table S3. Effect of NaOH initial concentration towards optical and kinetic properties.

C_{NaOH} (M)	pH	k_1 (s^{-1})	k_2 (M^{-1} s^{-1})	λ_{max} (nm)
1	12.4	5.083E-4	1.939	608
0.5	11.6	1.124E-4	1.170	600
0.25	10.9	6.650E-5	0.735	592
0.175	10.5	4.089E-5	0.315	586
0.1	9.9	3.084E-11	0.279	576

Curves for the derivative of experimental Abs_t data, showing the rate of change in absorbance at λ_{max}. These curves show a maximum value when the rate of growth at the steep increase in absorbance has the maximum value. These curves allow to retrieve information about the kinetic profile of the formation of AuNPs colloidal suspensions.

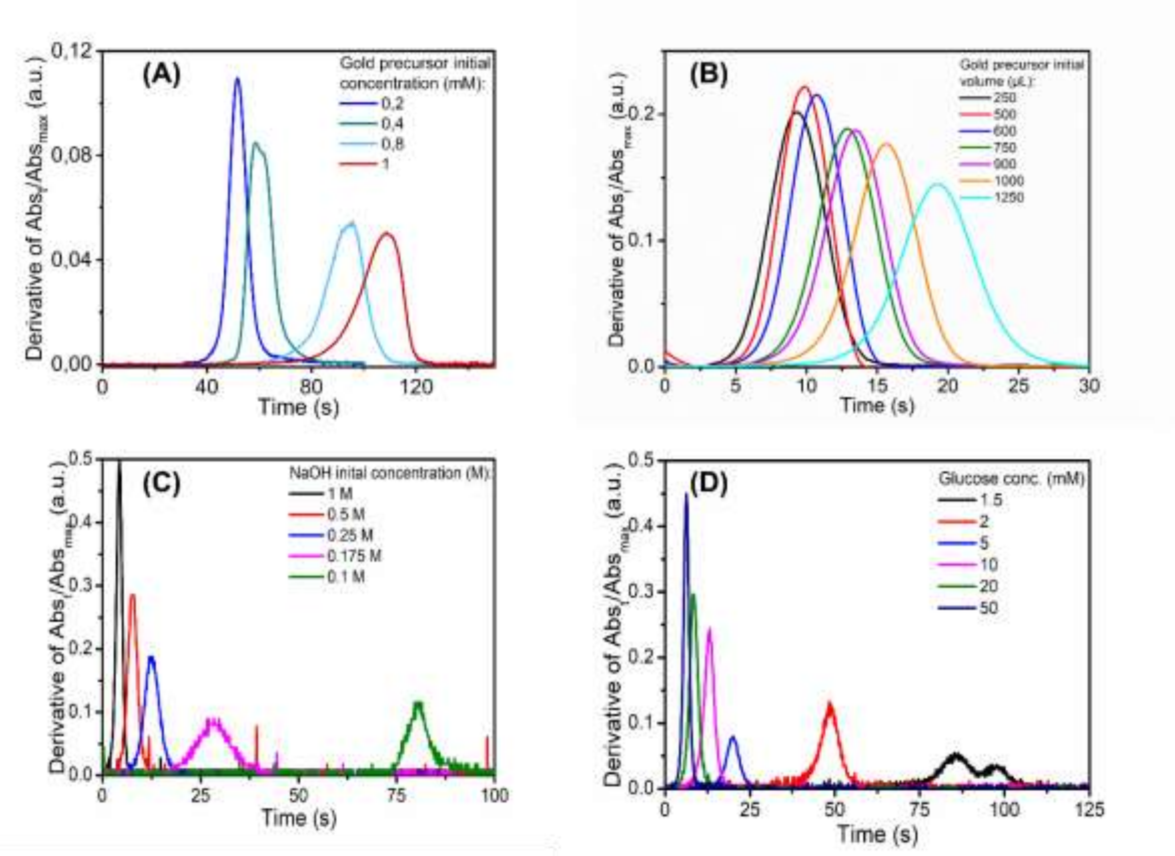

Figure S2. Curves for the derivative of experimental Abst data. (A) Derivative of experimental curves for the study of gold salt concentration effect towards kinetic properties. (B) Derivative of experimental curves for the study of gold salt volume effect. (C) Derivative of experimental curves for the study of NaOH concentration/pH effect. (D) Derivative of experimental curves for the study of glucose concentration effect.

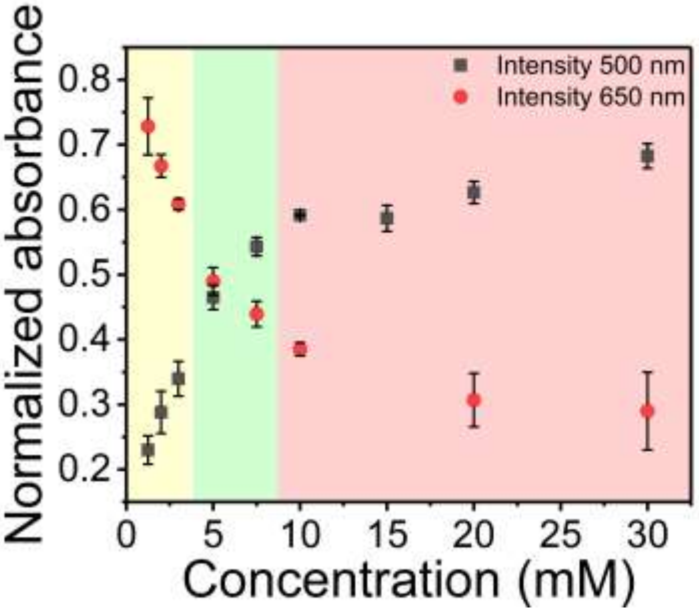

Figure S3. Correlation of normalized absorbance at 500 and 650 nm towards glucose concentration, showing that for a wavelength bellow the interval of resonance peak wavelengths, there is a correlated increase of intensity with the increase of glucose concentrations, with the opposite being observed for the normalized absorbance at 650 nm.

Assay performance for $HAuCl_4$ concentration of 10 mM and NaOH concentration of 1M, showing the effects of increasing molar concentrations of glucose to the colorimetric behavior of AuNPs synthesized at paper surface. As it can be noted, the particles give the paper in the reaction zone a pink hue, that increases it intensity for higher glucose concentrations. Given this result, the color intensity was monitored through the RGB color space, more specifically the grey scale intensity (mean of Red, Green and Blue channels), to achieve optimized color signals for correlation with glucose concentrations.

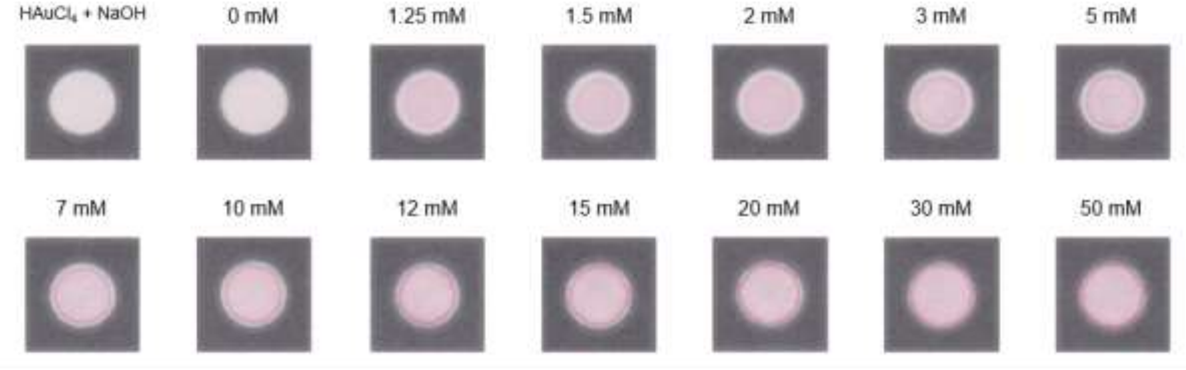

Figure S4. Glucose detection approach in paper substrate. As higher glucose concentrations are applied, a more intense color signal if produced, which can be correlated with glucose concentration through digital analysis of various color spaces.

Study of colorimetric signals produced using different reaction parameters (10, 20 and 30 $HAuCl_4$ concentrations and 1, 2 and 3 M NaOH concentrations) was performed, using two different metrics for the digital analysis. In **Section 2.2.1**, the histograms for the color intensity measured using the grey scale intensity are presented, while

in **Figure S4**, the same analysis is presented for the Red x Value metric, where we achieved similar results for the choice of the optimized reaction conditions (30 mM $HAuCl_4$ and 3 M NaOH concentration).

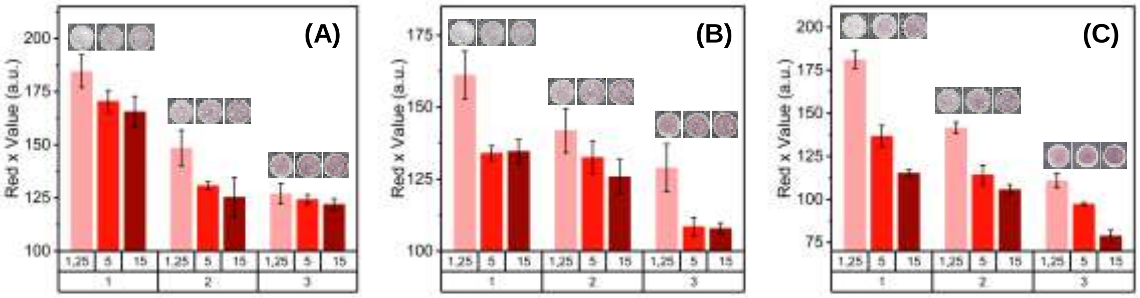

Figure S5. Optimization of colorimetric signals for the paper-based glucose assay. (A) Hystograms of RedxValue metric comprison for 10 mM HAuCl4 concentration and three glucose concentrations (1.25, 5 and 15 mM). (B) Comparison for 20 mM HAuCl4 concentration. (C) Comparison for 30 mM HAuCl4 concentration.

Color signal stability study was performed, to determine the optimal time to perform paper platform scanning, to extract colorimetric features. Using the mean grey scale intensity in crops and making the comparison between the intensity for the lowest (1.25 mM) and highest (20 mM) tested glucose concentrations (color difference in **FIGURE S5**), we showed that color signals get more similar with time, being harder to distinguish glucose concentrations in different levels. In the first 5 minutes, color signal range variation is higher and so, 2 minutes are established as the time for extraction of platform imaging.

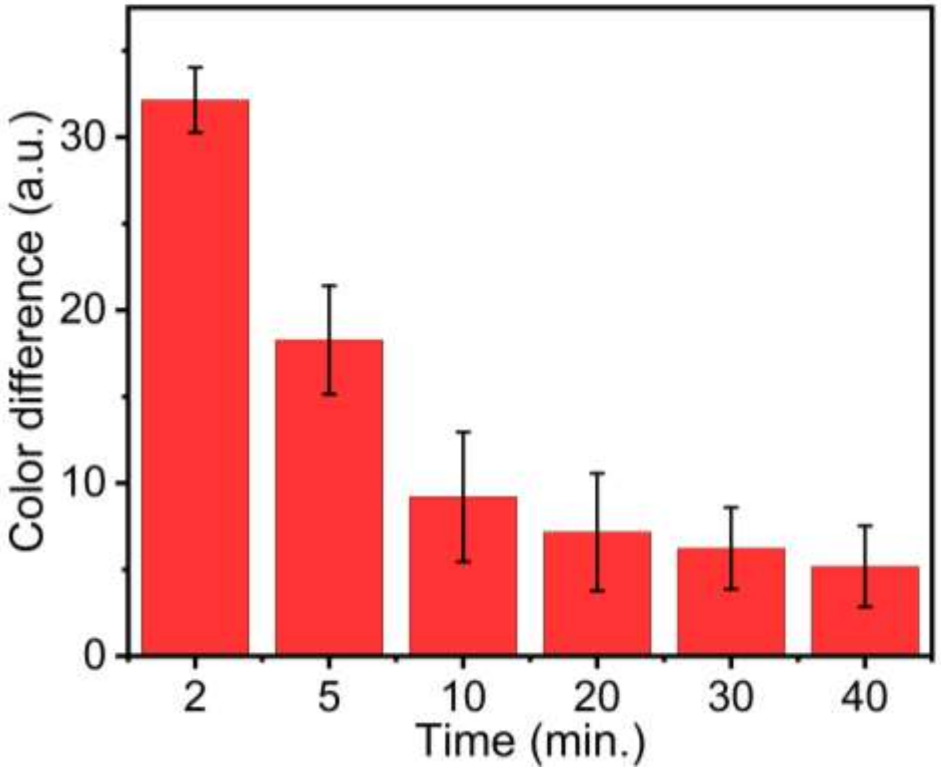

Figure S6. Histogram of color difference describing the stability of color signals in the paper-based assay. With time, the difference between color signals for the lowest and highest glucose concentration decreases, showing that the extraction of color features should be fixed and performed before 5 minutes after assay performance.

Table S4. Interference assay results from Figure 9A.

**Analyte**	**Greyscale Intensity (a.u.)**	**5 mM**	**Physiological concentrations**
Glucose		131.30 ± 2.16	119.89 ± 2.81
Fructose		157.08 ± 2.82	163.34 ± 1.98
Asc. Acid		132.37 ± 2.81	161.95 ± 2.78
Glutathione		164.18 ± 1.75	160.97 ± 3.59
Galactose		135.52 ± 3.07	159.67 ± 1.51
Lipoic acid		164.65 ± 2.17	161.11 ± 1.34

Table S5. Interference assay results from Figure 9B and S5.

Glucose concentration (mM)	Greyscale Intensity (a.u.)	Glucose	Glucose & Interfering agents	Red x Value (a.u.)	Glucose	Glucose & Interfering agents
1.5		166.69 ± 2.89	165.35 ± 3.30		119.20 ± 3.73	118.50 ± 5.88
2.5		148.52 ± 3.86	151.90 ± 1.13		103.02 ± 4.60	100.68 ± 1.06
5		131.30 ± 2.16	138.05 ± 1.22		87.63 ± 2.05	88.59 ± 1.53
10		125.81 ± 0.95	124.94 ± 1.60		79.61 ± 0.99	73.80 ± 2.14
15		119.89 ± 2.81	119.06 ± 1.97		67.91 ± 1.07	67.40 ± 1.68

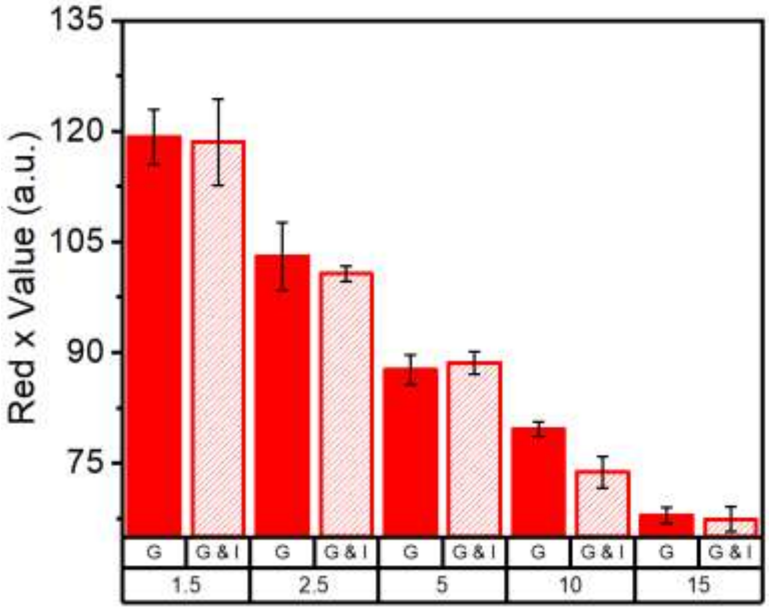

Figure S7. Interference assay for assessment of specificity towards glucose. Color signals obtained for samples simultaneously containing glucose and interfering agents (G + I), with concentration in the physiological levels, are compared with signals containing glucose individually (G), showing that there are no significant alterations in the color signals that may hinder the capability to distinguish different glucose concentration levels. The plot of correlation between grey scale value and glucose concentrations are presented in inset

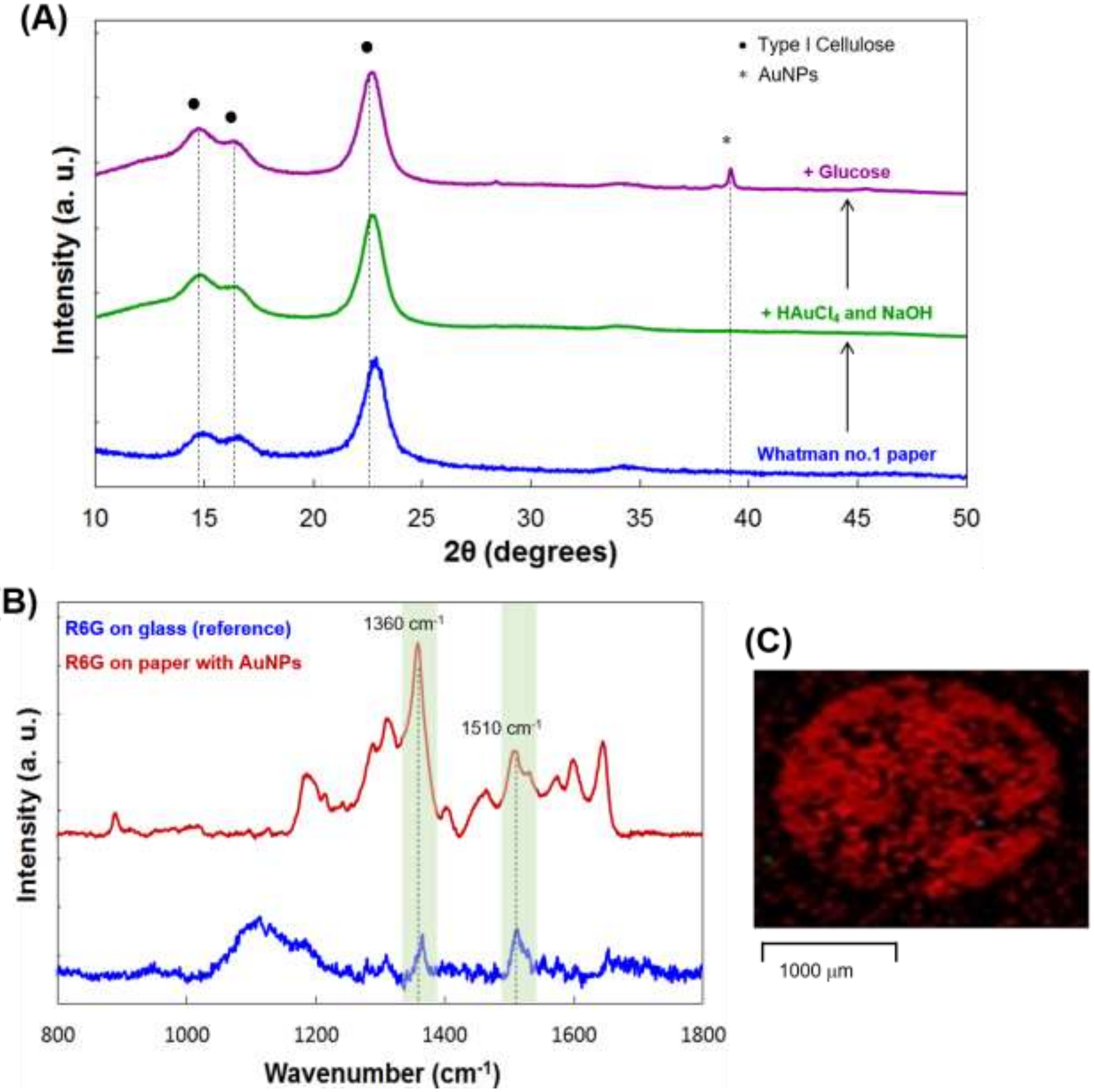

Figure S8. Characterization of the AuNPs synthesized on paper. (A) XRD diffractograms; (B) Comparison between the Raman signal from R6G on glass (reference substrate) and the SERS signal (enhanced) corresponding to R6G on paper with AuNPs; (C) Distribution of R6G molecules (red color) across the entire area of the well on the SERS-active substrate surface. The intensity of the red color is proportional to the 1510 cm-1 vibrational line intensity from R6G.

To have a figure of the efficacy of protein precipitation using ethanol, a spectrophotometric study was performed using the Biuret reagent, which reacts to the presence of protein by changing from a blue color to purple. Firstly, serum samples were diluted (50 to 0.01% serum samples) and were mixed with Biuret reagent (1:1 ratio in volume) to assess the response, establishing that for diluted samples above 1%, enough protein are present to induce the color change in the biuret reagent (**Figure S8A** and **B**). The plot of serum dilution *vs.* wavelength of the resulting mixture was performed and is shown in **Figure S8B**. Posteriorly, the same assay was performed for samples treated with ethanol, were precipitated proteins were previously removed. Results presented in

Figure S8D, showed that for a 1:3 serum to ethanol ratio, the protein removal is equivalent to diluting a serum sample 1000-fold (0.1% serum), with a resulting wavelength around 656 nm. Thus, a 25% serum solution obtained from the 1:3 serum to ethanol ratio results in approximately 1000-fold reduction in protein concentration, showing its efficacy.

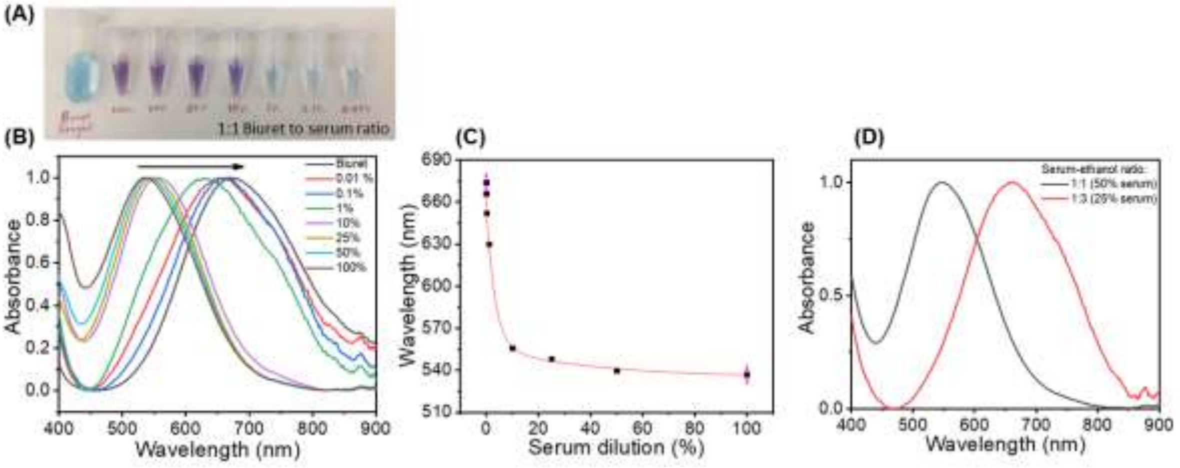

Figure S9. Study of protein precipitation and removal efficacy using ethanol for posterior paper-based AuNPs synthesis. (A) Color behavior of Biuret reagent towards the mixing with different fold-diluted serum samples. (B) Spectrophotometric profile of the obtained solution. (C) Plot of peak wavelength vs. serum dilution percentage. (D) Optical profile of Biuret reagent towards mixing with solutions treated with ethanol for protein precipitation (1:1 and 1:3 serum to ethanol ratio).
